# Reversible three-dimensional chirality continuum enabled by luminomagnetic superstructure in gel

**DOI:** 10.1038/s41467-026-73140-x

**Published:** 2026-05-11

**Authors:** Ki-Jae Jeong, Lulu Zhang, Fulin Jia, Fenglian Qi, Jianxiao Gong, Zhiyong Tang

**Affiliations:** 1 CAS Key Laboratory of Nanosystem and Hierarchical Fabrication, CAS Center for Excellence in Nanoscience, National Center for Nanoscience and Technology, Beijing, P. R. China; 2 Institute for Sciences of the Universe, Chungnam National University, Daejeon, Republic of Korea; 3 University of Chinese Academy of Sciences, Beijing, P. R. China; 4 Present Address: Key Laboratory of Optic-electric Sensing and Analytical Chemistry for Life Science, Ministry of Education, College of Chemistry and Molecular Engineering, Qingdao University of Science and Technology, Qingdao, P. R. China

**Keywords:** Nanoparticles, Self-assembly, Gels and hydrogels

## Abstract

Chirality continuum is crucial for advancing fundamental theories - spanning symmetry breaking and topological invariants - and for developing functional materials with tunable chiroptical, spintronic, and magnetic properties. However, achieving chirality continuum, particularly reversible in three-dimensional solids, remains elusive due to the intrinsic difficulties in nanoscale accuracy over extended architectures. Here, we introduce a decoupled design strategy that integrates magnetic field-directed achiral superstructures of luminomagnetic nanoparticles (LMNPs) with post-curing mechanical manipulation. Under a quadrupolar magnetic field, LMNPs self-assemble into nematic superstructures that are fixed within elastomers to form a luminomagnetic gel (LMG). Under macroscopic torsion, the superstructures undergo a transition from achiral to chiral nematic phase, allowing continuous and reversible tuning between left- and right-handed states. This process converts bulk mechanical deformation into nanoscale structural reconfiguration (for instance, 45° twisting in LMG produces 0.00088° of average inter-chain angle reorientation), thereby establishing long-range chiral ordering. The emergent circularly polarized luminescence is robust and reversible, with its intensity and handedness smoothly tuned through adjusting the applied torque. Our work delivers a modular, solid-state platform for reversible chirality continuum, opening avenues for polarization engineering and high-precision/broadband chiroptical technologies.

## Abstract (web summary)

Achieving chirality continuum, particularly reversible in three-dimensional solids, remains challenging due to the intrinsic difficulties in nanoscale accuracy over extended architectures. Here, the authors introduce a decoupled design that integrates magnetic field-directed achiral superstructures of luminomagnetic nanoparticles with post-curing mechanical manipulation.

## Introduction

Chirality, a pseudoscalar geometric attribute that forbids superposition of an object with its mirror image, can be continuously described in mathematical and theoretical frameworks^1^. The concept of chirality continuum, which spans the complete spectrum from left- to right-handed configurations, is of fundamental significance. It not only deepens theoretical studies in symmetry breaking and topological matter, but also drives practical applications in chiral devices, chiral metamaterials, chiral catalysis, etc^2–4^. The fabrication of the materials with the chiral architectures that can be tuned with arbitrary precision across this continuum is a prerequisite for high-resolution, broadband chiral metrology, dynamic chiral displays, and elucidation of structure-property relationships in chiral matter.

In natural systems, molecular chirality is inherently discrete, manifesting as one of two fixed enantiomers (levo- and dextro-), because its geometry is rigidly dictated by electron wave function eigenstates^5^. Bottom-up assembly of nano-building blocks offers an abundant degree of freedom for structural design compared with molecules^6,7^, allowing access, in principle, to a continuous chirality spectrum^2^. For instance, introducing DNA origami endows the switching between left- and right-handed nanoassemblies^8^, meanwhile partial tunability has been achieved in two-dimensional nanocomposites^3,4^ and chiral metasurface^9^. Yet these advances remain limited: the colloidal dispersions suffer from poor structural stability^10^ and are constrained to discrete switching between enantiomeric states^8^, whereas the chirality-tunable solid-state systems provide only incremental deformation within a single handedness^3,4,9^ (Supplementary Table 1). This grand challenge prompts us to explore how to obtain fine and reversible tuning across a full range of chirality - from left-handed, to achiral, to right-handed states - in three-dimensional (3D) solid-state systems.

One of the most readily accessible routes to nanoscale chirality is the arrangement of nanoparticles (NPs) into liquid crystal-like superstructures^11–14^. When NPs organize into long-range chiral nematic^14–17^ or smectic phases^13^, the collective chiroptical properties would emerge. However, the very dynamics that enable chiral tunability of the liquid crystal-like assemblies in solution also compromise their structural stability^11,17^. Embedding the NPs into elastic matrices can suppress Brownian motion and lock in long-range order^4,9,18–21^_._ Nevertheless, the predesigned chirality of these materials before polymerization makes macroscopic deformation of the elastomers usually insufficient to invert the intrinsic chirality^3,4,9^. We overcome this impasse by exploiting the continuum mechanics of nematic elastomers. Because both continuum fields and nematic order parameters can evolve smoothly in space and time^22–24^, the embedded achiral nematic NP superstructures can be driven through a continuous, symmetry-converting pathway from left- to right-handed configurations (or any state in between) within a single, solid-state material. This marriage of nematic assembly and elastic continuum thus transforms the discrete notion of chirality into a true, reversible 3D chirality continuum^2^.

## Results

### Design for LMGs with elastic chiral phase transitions

**Fig. 1:**
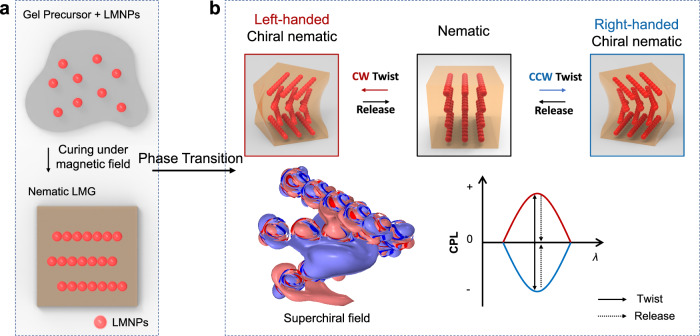
Elastic chiral phase transition of luminomagnetic gels (LMGs). **a** Scheme of constructing LMGs. Simultaneous curing of gels and magnetic assemblies results in the fixation of the nanoparticle superstructures in the elastic gels, which enables further chirality manipulation of the assembled nematic superstructures with structural stability. **b** Scheme of LMGs with three representative phases (nematic, left-handed chiral nematic, and right-handed chiral nematic) and corresponding chiroptical properties (superchiral field and circularly polarized luminescence (CPL)).

The formation of liquid crystal-like superstructures necessitates anisotropic building blocks to establish orientational order. This requirement is easily met by magnetic objects, since they tend to organize into anisotropic linear structures aligned along the magnetic flux, primarily driven by the magnetic dipole-dipole interactions^25^. This principle has been adopted for the preparation of colloidal photonic crystals^26^. However, the resulting superstructures are intrinsically unstable because field gradients exert attractive forces to ultimately pull the magnetic NPs toward the magnet^27^. In this study, we eliminate this instability by locking the luminomagnetic NPs (LMNPs) in place. An in-situ polymerization step cures the surrounding gel precursor during the magnetic assembly process (Fig. 1a). Since the external magnetic field is not inherently chiral, the resulting assembled structures are of an achiral nematic phase^17^. When the luminomagnetic gels (LMGs) are twisted, the macroscopic torsion breaks the mirror symmetry and continuously converts the nematic lattice into a chiral nematic (cholesteric) phase (Fig. 1b)^28^. The handedness and pitch of the induced chiral structures are dictated in real time by the direction and magnitude of the applied torque. Furthermore, upon release of torsion, the elasticity of LMGs restores the original achiral nematic order. This on-demand reconfiguration will yield ultra-fine tunability of chiral features, including superchiral field and circularly polarized luminescence (CPL) properties.

### Synthesis of LMNPs

**Fig. 2:**
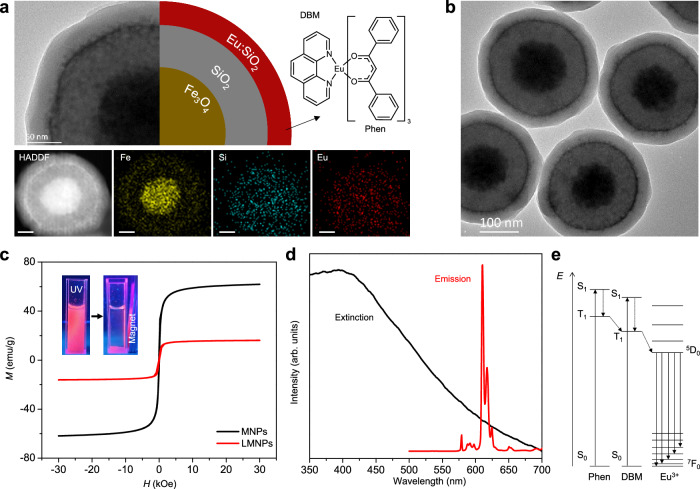
Eu-doped Fe_3_O_4_@SiO_2_ luminomagnetic nanoparticles (LMNPs). **a**, **b** Structural scheme, TEM image, and elemental mapping of LMNPs. Scale bars are 50 nm. **c** Magnetic hysteresis curves of Fe_3_O_4_ nanoparticles (MNPs) and LMNPs. The inset shows the magnetic separation of LMNPs under ultraviolet light (UV). **d** Extinction and photoluminescence (PL) spectra of LMNPs and **e** corresponding electron energy level diagram of tris(dibenzoylmethane) mono(1,10-phenanthroline) europium(III) (Eu(DBM)_3_(Phen)).

We began by synthesizing the building blocks, LMNPs, which combined bright, matrix-independent emission with rapid magnetic responsiveness (Fig. 2a). The Fe_3_O_4_ cores with an average diameter of 127.0 ± 8.3 nm were first prepared via a solvothermal method (Supplementary Fig. 1). Subsequently, the magnetic cores were coated with a silica shell with a thickness of 52.8 ± 3.7 nm using the Stöber method. This silica shell served as a separator to create sufficient distance between the magnetic core and the luminescence shell, effectively suppressed luminescence quenching^29^. A second 31.4 ± 2.2 nm-thick silica shell containing tris(dibenzoylmethane) mono(1,10-phenanthroline) europium(III) (Eu(DBM)_3_(Phen) complex) (Supplementary Figs. 2–4) was then deposited. Eu(DBM)_3_(Phen) complex offers not only a sharp, long-living emission that remains easily detected even in light-scattering elastomers, but also an emission wavelength that avoids spectral overlap with the absorption of Fe_3_O_4_ NPs^30^. Transmission electron microscope (TEM) images clearly display the triple-layer architecture, including magnetic core, spacer silica shell, and Eu(DBM)_3_(Phen)-containing silica shell, giving rise to the uniform LMNPs with an overall diameter of 294.3 ± 9.9 nm (Fig. 2b).

The bare Fe_3_O_4_ cores, (aggregates of 9.2 ± 2.9 nm single-crystalline Fe_3_O_4_ particles) display superparamagnetism with a saturation magnetization of 61.8 emu/g (Fig. 2c, Supplementary Fig. 5). After being coated with non-magnetic silica shells, the LMNPs retain the superparamagnetic property with a reduced saturation magnetization of 16.1 emu/g. The extinction spectrum shows that LMNPs have strong absorption near 400 nm and are almost transparent around 600 nm (Fig. 2d), ensuring that the Eu-complex emission remains unattenuated. Consequently, the LMNPs demonstrate intense, narrow-band photoluminescence (PL) at 610 nm (FWHM ≈ 10 nm) with a quantum yield of 4% and a lifetime of 0.38 ms, corresponding to the ^5^D_0_ → ^7^F_2_ transition (Fig. 2e, Supplementary Fig. 6). These magnetically responsive, highly emissive LMNPs thus serve as ideal multifunctional building blocks for magnetic field-direct assembly and collective chiroptical behaviors (Supplementary Fig. 7).

### Quadrupolar magnetic field directed nematic superstructure of LMNPs

**Fig. 3:**
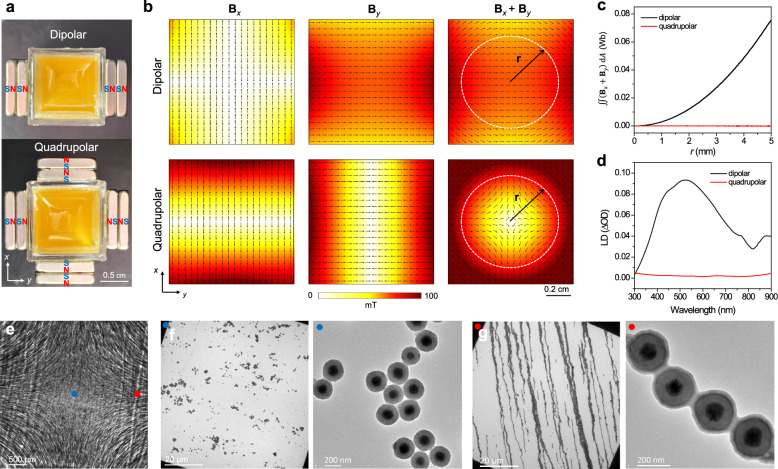
Quadrupolar magnetic self-assembly. **a** Photos of homemade setups for the dipolar and quadrupolar magnetic field-directed assembly. Magnetization direction of magnets is marked as N and S. **b** Magnetic field strength and direction mapping in the *x-y* plane of dipolar and quadrupolar arrays of magnets. **c** The area-integrated magnetic field in the circle with the radius **r** (**B**_*x*_ + **B**_*y*_ in Fig. 3b). **d** Linear dichroism (LD) spectra of the superstructures formed under the dipolar and quadrupolar magnetic field (without twisting). **e** A confocal microscope image of LMGs, which contains a topological defect at the center (shown as a blue dot). Transmission electron microscope (TEM) images of **f** topological defect at the center and **g** linear chains at the out-of-center (shown as a red dot in Fig. 3e).

The magnetic-field alignment of nano-objects often generates significant linear dichroism (LD), which would masquerade as or swamp the true circular dichroism (CD) signals and compromise optical reliability^15,18^. To avoid this, we opted for a quadrupolar array of magnets during the assembly process (Fig. 3a).

We compared the dipolar and quadrupolar magnet arrays (Fig. 3a, Supplementary Fig. 8) by simulating the in-plane strength and distribution of the magnetic field. The *x* and *y* components of the magnetic field (**B**_*x*_ and **B**_*y*_) are plotted using arrows, along with the combined one (**B**_*x*_ + **B**_*y*_) (Fig. 3b). In the dipolar array, the **B**_*x*_ pattern is mirror-symmetric under reflection about the *y*-axis, which cancel each other out in the vector sum. On the other hand, **B**_*y*_ remains positive in the net contribution throughout the assembly area due to the absence of the -axis mirror plane. Hence, the summation **B**_*x*_ + **B**_*y*_ is always positive along the *y*-axis, suggesting a unidirectional magnetic field. Conversely, the quadrupolar array generates **B**_*x*_ and **B**_*y*_ distributions that possess two orthogonal mirror planes with respect to the central axes. As a result, the **B**_*x*_ + **B**_*y*_ maintains higher-order mirror-symmetric, representing a non-unidirectional geometry to offset each other. To quantify this, we integrated **B**_*x*_ + **B**_*y*_ over circular areas of increasing radius **r** (Fig. 3c). For the dipolar array, the value of **B**_*x*_ + **B**_*y*_ rises exponentially with **r**, confirming that the collective directionality increases with the system size. For the quadrupolar array, the integral remains nearly zero and independent of **r**, implying the size-invariant, directionally compensated magnetic field.

This fundamental difference in the collective directionality of the magnetic field makes the quadrupolar magnet array an excellent choice to eliminate the LD signal. As observed in the LD spectra, the quadrupolar assembly shows negligible absorption in a broad wavelength range (Fig. 3d, Supplementary Fig. 9). Quantitatively, the LD intensity drops from 0.0793 ΔOD with the dipolar assembly to 0.0016 ΔOD with the quadrupolar one, a 98% reduction that reflects the globally isotropic alignment obtained with the latter configuration. Therefore, the non-unidirectional geometry of the quadrupolar magnetic field can effectively eliminate the formation of unidirectional superstructure, which is the origin of strong LD in the dipolar magnet array directed system.

3D LMGs with a size of 1 × 1 × 2 cm were made with a simple homemade setup (Fig. 3a). After curing, solid-state luminescent LMGs were formed. To investigate the 3D characteristics of the magnetic field, we simulated the 3D magnetic field distribution and flux within the LMG space (Supplementary Fig. 10). Side-view analysis demonstrates that the magnetic flux remains orthogonal to the *z*-axis throughout the sample volume, indicating excellent field uniformity. This is further supported by confocal *z-*stack images showing identical alignment in different *x-y* planes (Supplementary Fig. 11). Under the reflected light, they exhibit a characteristic brush texture (Supplementary Fig. 12) due to the disclination (strength of disclination *s* = −1). The confocal microscope image reveals that the microscopic pattern of the as-assembled superstructures displays four-fold symmetry (Fig. 3e), mirroring the underlying quadrupolar field. A topological defect, where the net magnetic force is zero, appears at the pattern center (blue dot in Fig. 3e). In this topological defect region, LMNPs remain isotropic (Fig. 3f), in stark contrast to the uniform linear chains produced under a dipolar field (Supplementary Fig. 13). We also notice that these linear chains appear at the periphery of the quadrupolar assembly (Fig. 3g). Altogether, the overall texture of LMGs is nematic, validating that the LMNPs assemble into a nematic phase within the LMGs.

### Elastic chiral features of LMGs

**Fig. 4:**
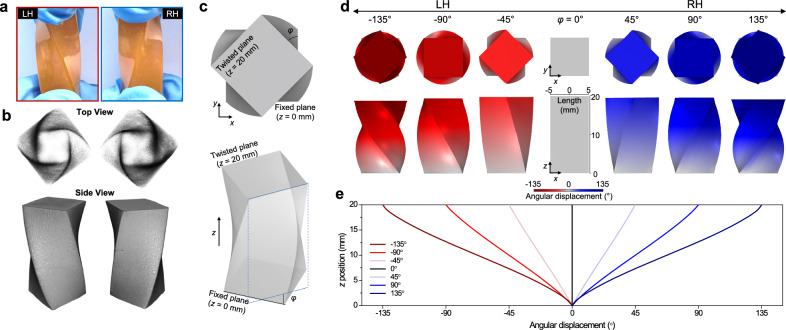
Mechanical deformation of LMGs. **a** Hand-twisting LMGs in left-handed (LH) and right-handed (RH) configurations, and **b** corresponding computed tomography (CT) images. **c** Scheme of twisting with designed angle *φ*. **d** Mechanical deformation simulations of the elastomers depending on *φ*, and **e** corresponding angular displacement plot along the twist axis.

The LMGs assembled under the quadrupolar field are composed of numerous layers whose in-plane patterns (Fig. 3e) are replicated along the *z*-axis. When torsion is applied, the elastic LMGs translate macroscopic twist into a smooth rotation of these layers along the twisting axis (*z-*axis) (Fig. 4a). 3D computed tomography (CT) images reveal the dark spiral patterns, evidencing the continuous rotation of the assembled structures (Fig. 4b). Even after repeated twisting, the structures maintained their linear configurations without breakage (Supplementary Fig. 14).

Furthermore, the handedness and pitch of the helix are governed continuously by *φ*, the angle between the bottom fixed plane and the top twisted plane (Fig. 4c, d, Supplementary videos 1, 2). It deserves noting that over the entire torsional strain range, −135° ≤ *φ* ≤ 135°, the LMGs exhibit characteristic of linear elastic properties (Supplementary Figs. 15–17). The gradual change in the angular displacement along the twisting axis is depicted by the continuous lines in Fig. 4d, e, resulting in a uniform inter-chain twist angle in the nematic superstructures. This, in turn, leads to the emergence of a long-range, uniformly chiral nematic phase.

### Elastic CPL of reversible chirality continuum

**Fig. 5:**
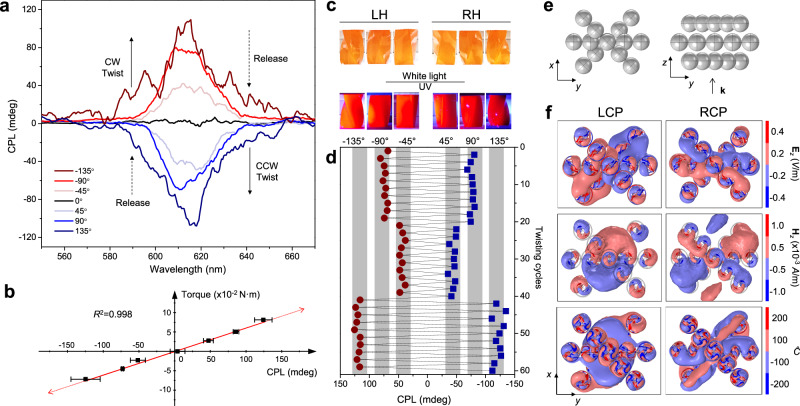
Elastic circularly polarized luminescence (CPL) of LMGs. **a** CPL spectra depending on the twisting direction and degree. **b** Correlation between torque and CPL intensity. **c** Photos of LMGs under white light and UV, and **d** corresponding CPL peak intensity in multiple reversible twisting cycles. **e** Geometry used in the optical simulation (left-handed twisted-nanochains with the inter-chain angle of 45°). The direction of light propagation is in the +*z* direction. **f** Visualization and comparison of the electric field (**E**_*z*_), the magnetic field (**H**_*z*_), and the superchiral field (*Ĉ*) in the vicinity of twisted nanochains, induced by left- and right-handed circularly polarized light (LCP and RCP), respectively, at the emission wavelength of 610 nm with the incident direction of the *z*-axis.

The untwisted nematic LMGs display no CPL signal, confirming the achirality of the assembled superstructures (Fig. 5a, Supplementary Figs. 18–20). Upon torsion, a CPL peak at 610 nm emerges, coincident with the emission peak of the LMNPs (Fig. 2d). Evidently, the use of a rare-earth emitter results in a narrow bandwidth (~20 nm) of the CPL peak. In addition, we systematically investigated the effects of nanoparticle concentration and magnetic-field intensity during assembly (Supplementary Fig. 21). Optimization of these parameters is crucial for optimal CPL performance. Moreover, both the sign and intensity of the CPL peak can be continuously reconfigured by varying the degree and direction of twisting. The dissymmetry factor (*g*-factor) reaches 0.0083 at *φ* = −135° and −0.0068 at *φ* = 135° (Supplementary Fig. 22). Thanks to the robust structural stability and linear elasticity of LMGs, torque and CPL are interconverted in a fully reversible fashion (Fig. 5b). The odd symmetry in the correlation between torque and CPL highlights the handedness conversion of LMGs.

In order to determine the possible LD effect induced by torsion, we characterized the LD under mechanical twisting (Supplementary Fig. 23). Regardless of *φ*, LD remains at a very low level (<0.02), confirming that LD contributions are negligible and do not influence the observed chiroptical responses. Other factors, such as illumination direction and matrix effect, showed similar results (Supplementary Figs. 24, 25). Additionally, LMGs prepared without a magnetic field produce no distinct chiroptical response and tendency upon twisting (Supplementary Fig. 26), confirming that the field-assembled nematic phase is indispensable for mechano-tunable chiroptical properties. Repeated torsion tests uncover that the CPL signal persists almost unchanged over tens of twisting cycles (Fig. 5c, d) and remains stable for more than one year (Supplementary Fig. 27), demonstrating the exceptional robustness of the nematic superstructures locked within the elastic matrix.

The generality of our strategy was evaluated using luminomagnetic nanorods, which also exhibit twist-induced CPL (Supplementary Fig. 28). Furthermore, we prepared a Tb-based LMG, which exhibits green emission (~575 nm) and clear twist-induced CPL (Supplementary Fig. 29). This confirms that the LMG platform is not limited to nanoparticle geometry or a specific emission wavelength.

To elucidate the origin of CPL in twisted LMGs, we performed light-matter interaction simulations for the twisted nanochain architecture to evaluate its optical response to left- and right-handed circularly polarized light (LCP and RCP) (Fig. 5e, Supplementary Fig. 30). The ensemble of magnetic and dielectric core-shell heterostructures brings strong spatial overlapping between the electric and magnetic components of resonance, resulting in a pronounced superchiral field^9,31,32^ in the vicinity of twisted nanochains (Fig. 5f, Supplementary videos 3, 4 and Supplementary Fig. 31). Because the Fe₃O₄ cores in LMNPs are magnetically active, the magnetic component induced by left- and right-handed circularly polarized light (LCP and RCP) differs more significantly than the electric counterpart^33^. Therefore, the superchiral field surrounding the twisted nanochains exhibits a pronounced asymmetry between LCP and RCP incidence. The twisted LMG acts as a macroscopically chiral and anisotropic optical medium with distinct responses to LCP and RCP components. As a result, achiral luminescence propagating through it undergoes differential extinction of its LCP and RCP components, converting the emission into an apparent CPL signal. The resulting optical asymmetry of the superchiral field increases monotonically with the inter-chain angle up to ~40° (Supplementary Fig. 32). Although the macroscopic twist imposed on the LMGs translates to average inter-chain angle as small as 0.00088°, 0.00176°, and 0.00264° for *φ* = 45°, 90°, and 135°, respectively (Supplementary Fig. 33), the centimeter-scale, liquid-crystal-like long-range order amplifies the chiral optical response. Consequently, the system delivers robust and measurable chiroptical properties (Fig. 5a, Supplementary Figs. 34–36) that are ready for scaling well beyond the current experimental window.

## Discussion

By embedding a quadrupolar magnetic field-induced, achiral nematic superstructure into an elastic matrix, we realize a solid-state chirality continuum that seamlessly spans left- to right-handed configurations via an achiral phase under simple, reversible torsion. The nematic liquid crystal-like superstructures with long-range order endow a strong chiroptical response even when inter-chain angles are minute. Unlike conventional systems that rely on pre-imprinted chirality, LMGs offer a platform for real-time, repetitive tuning of 3D structural chirality with no hysteresis, generating the mirror-symmetric CPL whose sign and magnitude respond instantly to torque direction and amplitude. Moreover, the linear CPL-torque correlation encodes the LMGs as continuous variables in a high-dimensional functional space, where geometry, mechanics, and optics coexist and interconvert. Chirality is thus redefined from a static geometric label to a dynamic, multidimensional continuum that evolves continuously in space, time, and correlated physical dimensions.

Leveraging these properties, LMGs demonstrate exceptional processability and environmental robustness. Their dynamic chirality continuum establishes them as cornerstone materials for reconfigurable, solid-state chiral devices. By translating continuous mechanical inputs into precise optical outputs, they enable non-destructive, in-situ readout and control of soft-matter microstructures, an advance poised to impact optical communications, dynamic holography, adaptive smart materials, and multifunctional interfaces for soft robotics and responsive biomedical sensors^34^.

## Methods

### Chemicals

Polydimethylsiloxane (PDMS, Sylgard 184) was purchased from Dow Corning Inc. Ferric chloride hexahydrate (FeCl_3_·6H_2_O), sodium acetate (CH_3_COONa), trisodium citrate dihydrate, ethylene glycol (HOCH_2_CH_2_OH), nonionic surfactant IGEPAL CO-520, and tetraethyl orthosilicate (TEOS) were bought from Sigma-Aldrich Ltd. Ammonium hydroxide (25% aqueous solution of NH_3_·H_2_O) and ethanol were obtained from Beijing Chemistry Reagent Company. Eu(DBM)_3_(Phen) was achieved from Shanghai Macklin Biochemical Co., Ltd. Deionized (DI) water (> 18 MΩ·cm^−1^) was used for all experimental procedures. All chemicals were used without further purification.

### Synthesis of LMNPs

1.3 g of ferric chloride hexahydrate was dissolved into 40 mL of ethylene glycol under magnetic stirring, followed by the addition of 2.4 g of sodium acetate and 1.2 *g* of trisodium citrate dihydrate. As-prepared solution was transferred to a Teflon tube (100 mL volume) and sealed in a stainless-steel autoclave. The solvothermal reaction proceeded at 210 °C for 12 h. The dark precipitates (Fe_3_O_4_ NPs) were washed with ethanol and water three times, respectively, followed by drying in the vacuum oven at 60 °C for 12 h.

5 mg of dried Fe_3_O_4_ NPs powder was redispersed in 9.5 mL of ethanol and 0.5 mL of DI water mixture with sonication. 1.2 mL of ammonia solution and 50 μL of tetraethyl orthosilicate were added in order. The solution was placed in the shaking oven at 300 rpm for 12 h. The silica-coated Fe_3_O_4_ NPs were washed with ethanol three times and stored as a 5 mg/mL colloidal solution in ethanol.

30 mg of Eu(DBM)_3_(Phen) was dissolved in 10 mL of ethanol and 5 mL of IGEPAL CO-520 mixture. The silica-coated Fe_3_O_4_ NPs stock solution (5 mg/mL) was diluted in ethanol (0.5 mg/mL). These two prepared solutions were mixed, followed by the addition of 1.2 mL of ammonia solution and 50 μL of TEOS, and sonicated for 1 h with a mild vortex. The final product was washed with ethanol and water three times.

### Fabrication of LMGs

5 mg of as-prepared LMNPs were dispersed in 2 *g* of PDMS curing agent, followed by mixing with 3 *g* of PDMS base. Mixed PDMS was poured into a 4.5 mL plastic cuvette and cured at 40 °C for 2 h under the quadrupole magnetic field. Specifically, neodymium magnets (Nd_2_Fe_14_B, *L × W × T* = 50 *× *10 *× *2 mm) were placed on the four sides of a plastic cuvette, with the same magnetic poles facing each other and with the opposite magnetic poles adjacent to each other. It was worth noting that temperatures above 60 °C should be avoided to allow sufficient time for the LMNPs to assemble and to preserve the magnetic properties of the magnets.

### Characterizations

A UV-Vis spectroscopy (Evolution 220, Thermo Scientific, Waltham, MA) was used to record absorption spectra. Transmission electron microscope images were acquired with a Tecnai G2 20 S-TWIN (FEI Company, USA), and HADDF STEM images were attained with a Tecnai G2 F20 U-TWIN (FEI Company, USA). The fluorescence images of the aligned NPs were taken with a confocal microscope (UltraVIEW VoX, PerkinElmer Inc., Waltham, MA, USA). A CD spectrophotometer (J-1500, Jasco Inc., Japan) and CPL spectrophotometer (CPL-200, Jasco Inc., Japan) were utilized to monitor the chiroptical properties.

### Computational simulation

The mechanical deformation and optical simulations were performed using the finite element method (FEM) implemented in COMSOL Multiphysics (COMSOL Inc., Boston, MA, USA). Material parameters, including the elasticity of PDMS and the refractive indices of Fe_3_O_4_, silica, and PDMS, were adopted from the software’s built-in materials library. Circularly polarized light was modeled by the superposition of two orthogonal linearly polarized lights (*x-* and *y-*component with ± *λ*/4 phase shift of *y-*components (for *z-*axis incident light). Simulation space was surrounded by a perfectly matched layer (perfect absorbing boundary conditions). The superchiral fields (*Ĉ*) were calculated using the following expression:

1 $$C=-\frac{{\omega \varepsilon }_{0}}{2}{{{\rm{Im}}}}\left[{{{{\rm{E}}}}}^{*}\cdot {{{\rm{H}}}}\right],$$

where **E** and **H** represent the complex electric and magnetic field, respectively, *ω* is the angular frequency of the incident light, and ${\varepsilon }_{0}$ is the vacuum permittivity. To quantify the chiral enhancement, the local superchiral field was normalized to that of a circularly polarized plane wave in free space (*C*_*0*_). Regions where the normalized enhancement factor *Ĉ* = |*C*/*C*_*0*_ | > 1 indicate the generation of superchiral light of the twisted nanochain structures.

## Supplementary information

Supplementary Information

Description of Additional Supplementary Files

Supplementary Video 1

Supplementary Video 2

Supplementary Video 3

Supplementary Video 4

Transparent Peer Review file

## Source data

Source Data

## Data Availability

The data generated in this study are provided in the Source data. The data are also available from the corresponding author upon request. Source data are provided with this paper.
