# Supplementary material for: Reversible three-dimensional chirality continuum enabled by luminomagnetic superstructure in gel
Source: Nat Commun. 2026 May 11;17:6319. doi: 10.1038/s41467-026-73140-x (PMC13377049; doi:10.1038/s41467-026-73140-x)
Supplement: Supplementary file 1 — Supplementary Information [file 41467_2026_73140_MOESM1_ESM.pdf]

Supplementary Information

Reversible Three–dimensional Chirality Continuum Enabled by Luminomagnetic Superstructure in Gel

Ki-Jae Jeong[1, 2], Lulu Zhang[1], Fulin Jia[1], Fenglian Qi[1, 4], Jianxiao Gong[1, 3*], Zhiyong Tang[1, 3*]

[1]CAS Key Laboratory of Nanosystem and Hierarchical Fabrication, CAS Center for Excellence in Nanoscience, National Center for Nanoscience and Technology, Beijing 100190, China

[2]Institute for Sciences of the Universe, Chungnam National University, Daejeon 34134, Republic of Korea

[3]University of Chinese Academy of Sciences, Beijing 100049, China.

[4]Current affiliation: Key Laboratory of Optic-electric Sensing and Analytical Chemistry for Life Science, Ministry of Education, College of Chemistry and Molecular Engineering, Qingdao University of Science and Technology, Qingdao 266042, China.

Correspondence to: zytang@nanoctr.cn, gongjx@nanoctr.cn.

Supplementary Table 1|Comparison with related researches

Ref.	Sample form	Chirality range	Reversibility	Continuity		Coupled parameter	Long–term stability
				Spatial	Temporal		
1	2D Solid	Single handedness	O	O	O	Mechanical	N/A
2	3D Solid	Single handedness	O	X	X	Magnetic field	N/A
3	2D Solid	Single handedness	O	O	O	Mechanical	N/A
4	2D Solid	Single handedness	O	O	O	Mechanical	N/A
5	3D Solid	Full	X	O	X	Chemical	N/A
6	3D Colloid	Full	O	O	O	Magnetic field	N/A
This work	3D Solid	Full	O	O	O	Mechanical	Semi–permanent

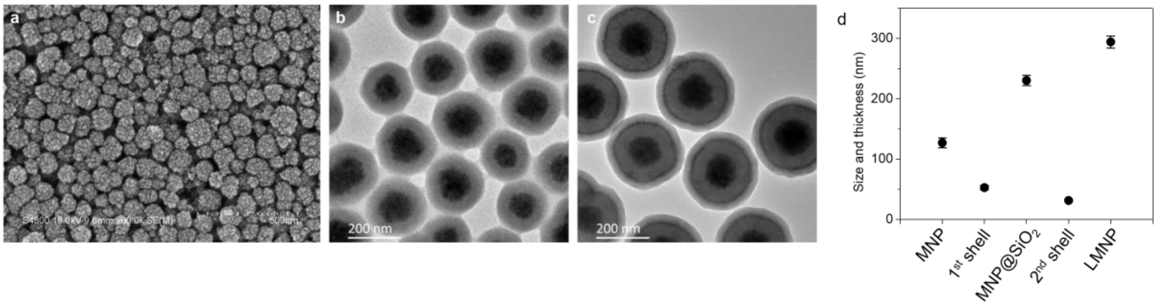

Supplementary Fig.1|Electron microscope images of (a) MNPs, (b) MNP@SiO_2, and (c) Luminomagnetic nanoparticles (LMNPs), and (d) corresponding size and shell thickness distribution plot. The average nanoparticle size is 127.0 ± 8.3 nm for (a) MNPs, 230.5 ± 8.3 nm for (b) MNP@SiO_2, and 294.3 ± 9.9 nm for (c) LMNPs.

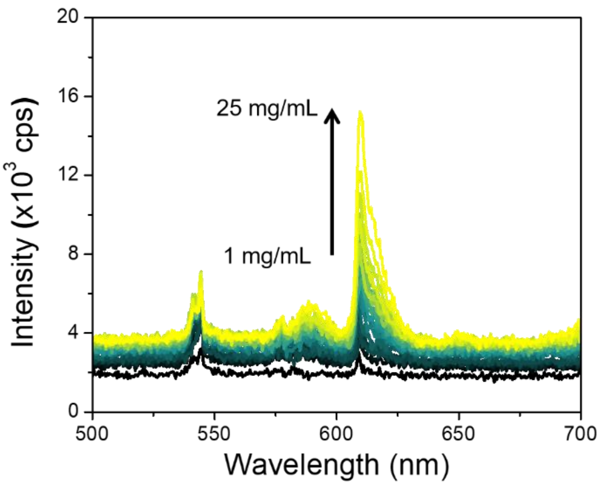

Supplementary Fig.2|Photoluminescence (PL) spectra of LMNPs depending on the concentration of $Eu(DBM)_3(Phen)$ complex. The intensity of PL increases with the concentration of Eu complex.

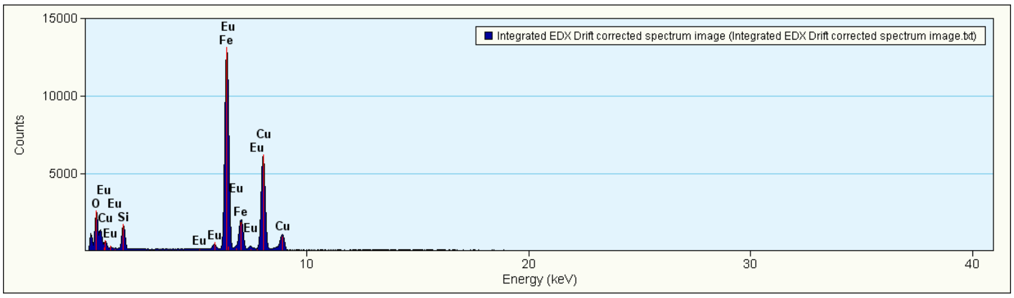

	Weight %	Atomic %
O (K)	14.47	36.75
Si (K)	4.77	6.91
Fe (K)	75.47	54.91
Eu (L)	5.27	1.41

Supplementary Fig.3|Energy–dispersive X–ray Spectroscopy (EDX) survey and corresponding element ratio of LMNPs.

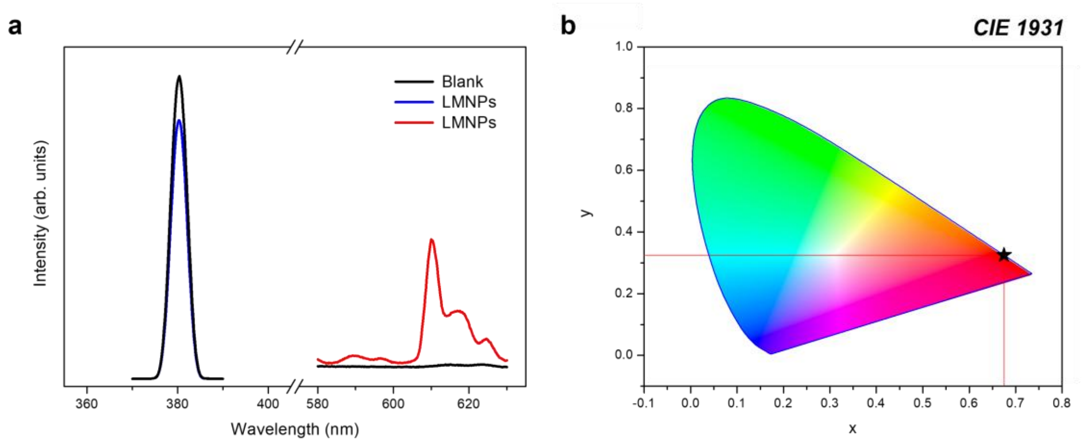

Supplementary Fig.4|Luminescence properties of LMNPs. (a) PL spectrum of LMNPs solution in the excitation and emission region. (b) CIE chromaticity diagram of LMNPs. The emission color was marked in black star. Quantum yield of LMNPs in water is ca. 4%.

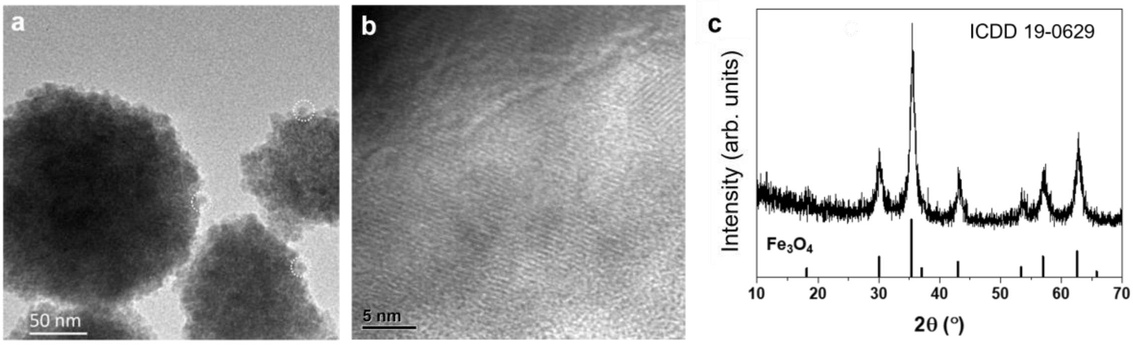

Supplementary Fig.5|(a) TEM and (b) High–resolution TEM (HRTEM) images of MNPs, and (c) X–ray diffraction (XRD) pattern of LMNPs. The mean size of the ordered crystalline domains of MNPs is estimated to be 9.2 nm, based on XRD peak width by the Scherrer equation.

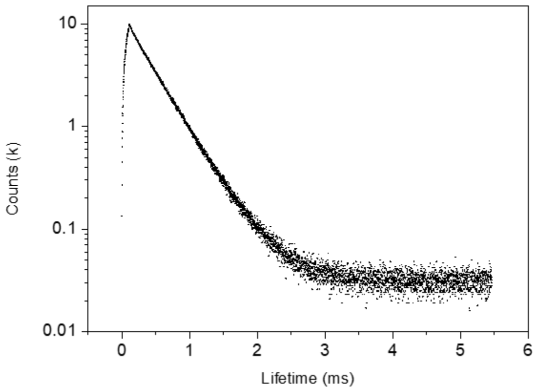

Supplementary Fig.6|Lifetime curve of LMNPs. The lifetime of LMNPs is measured to be 0.38 ms.

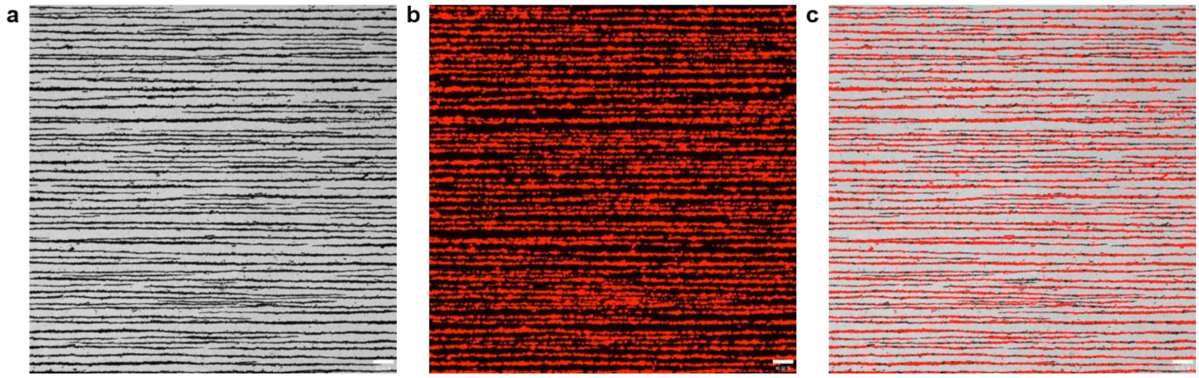

Supplementary Fig.7|Confocal microscope images of magnetically assembled LMNPs. (a) Bright field, (b) luminescence, and (c) merged image. Scale bars are 40 µm. The luminescence patterns exactly follow the assembled nanochains, indicating efficient assembly of LMNPs under a magnetic field.

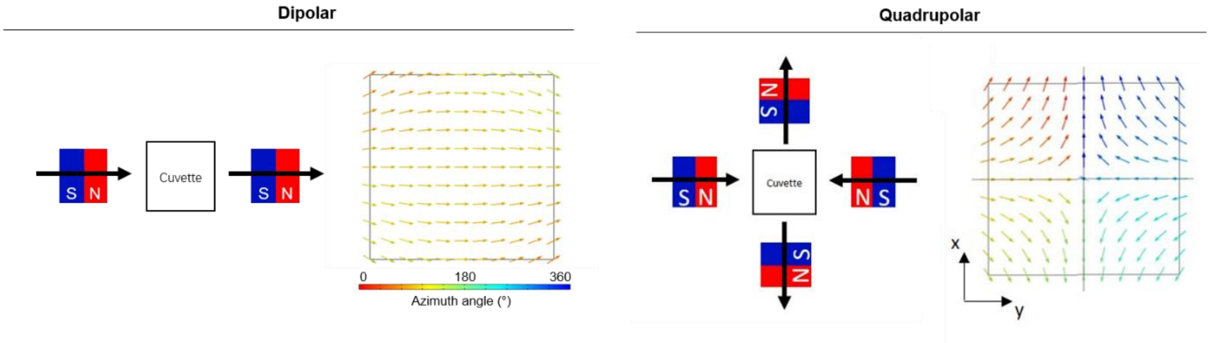

Supplementary Fig.8|Simulation result of magnetic field geometry of dipolar and quadupolar array of magnets. Scheme of magnets' position and corresponding magnetic field geometry at the cuvette region. The direction of the magnetic field at each point is displayed in both arrows and colors. The color legend shows the azimuth angle. The magnetic field geometry across the assembly area is simulated and depicted using the arrows to illustrate the distribution of the magnetic field vectors in the *x*–*y* plane. The color of the arrow at each position represents the azimuth angle of the magnetic field at that location. It is observed that the magnetic field direction of the quadrupolar magnet array encompasses all azimuth angles (0°– 360°) around the center, revealing a distinct magnetic field configuration from that of the dipolar magnet array.

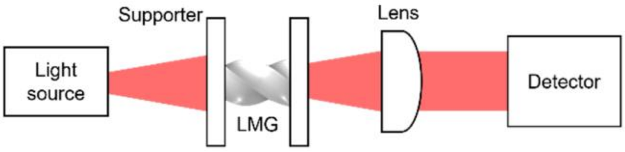

Supplementary Fig.9|Schematic illustration of the optical measurement setup in CD spectrophotometer for measuring CD and LD. The CD and LD were measured in ordinary CD spectrophotometer setup whose light source, sample, and detector is in a row. Unless otherwise specified, these experimental configurations were employed for all measurements.

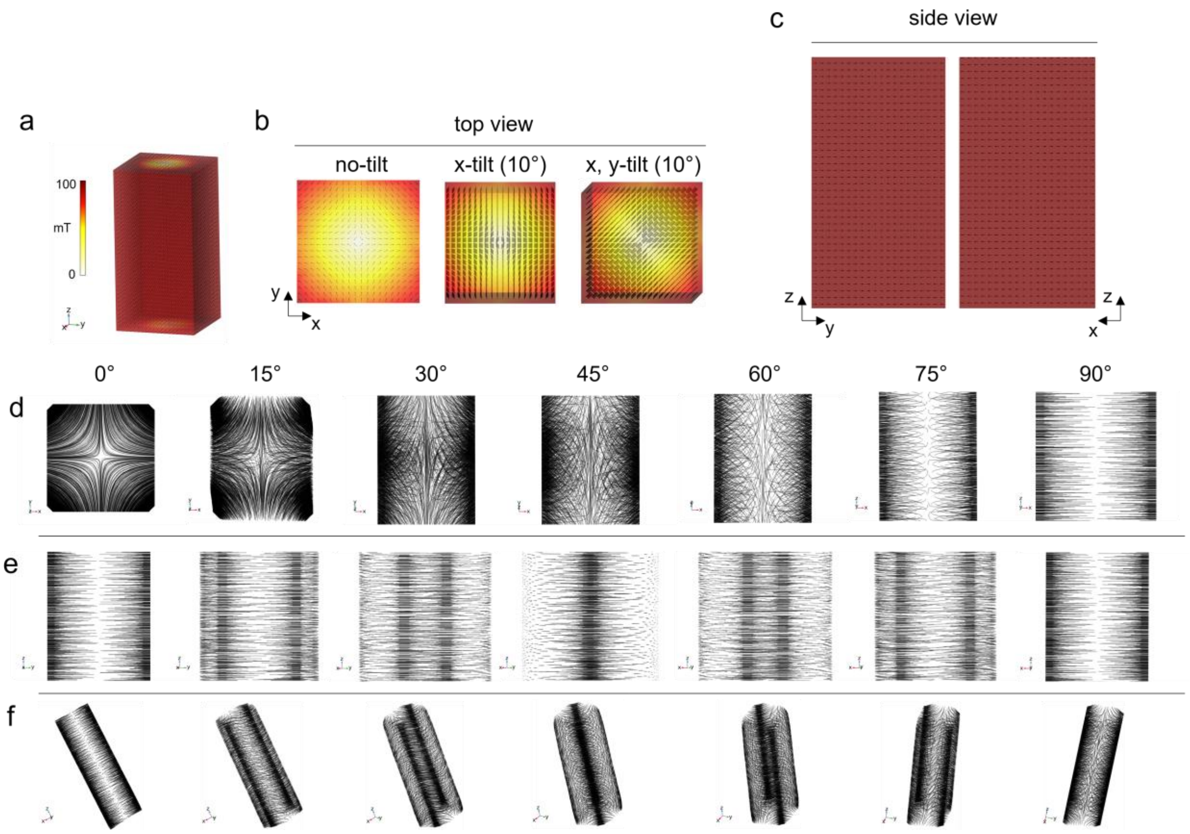

Supplementary Fig.10| Simulated 3D magnetic field distribution within the LMG space under the quadrupolar magnetic–field configuration. (a) Perspective view of the 3D magnetic field distribution within LMG space. (b) Top view of the magnetic field distribution with 0º tilting (no–tilt), 10º tilted view along *x*–axis (*x*–tilt) and 10º tilted view along *x*–axis and *y*–axis (*x*–*y*–tilt). (c) Side view of the magnetic field distribution. Magnetic field intensity and direction is presented as color and arrows. (d–f) Visualized magnetic flux images during rotation with different views. (d) Top view of magnetic flux upon rotation with *x*–axis. (e) Side view of magnetic flux upon rotation with *z*–axis. (f) Perspective view of magnetic flux upon arbitrary rotation axis.

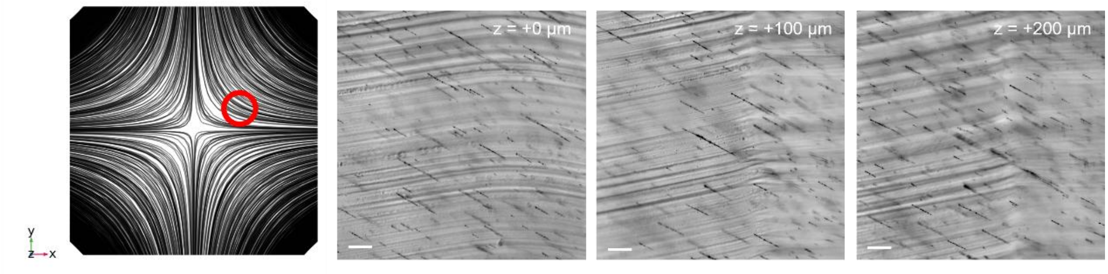

Supplementary Fig.11| Confocal microscope image with different *z*–position. Scale bars are 20 μm. Red circle in first scheme shows the *x*–*y* position where the confocal microscope image is taken.

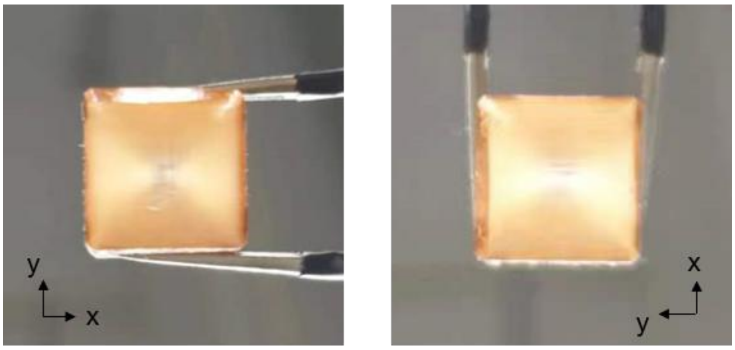

Supplementary Fig.12|Photographs of the LMG cross–section under light. Brush texture rotates continuously as the LMG turns, but it rotates twice as fast as LMG itself. Namely, the brush texture rotates π, while LMG rotates π/2.

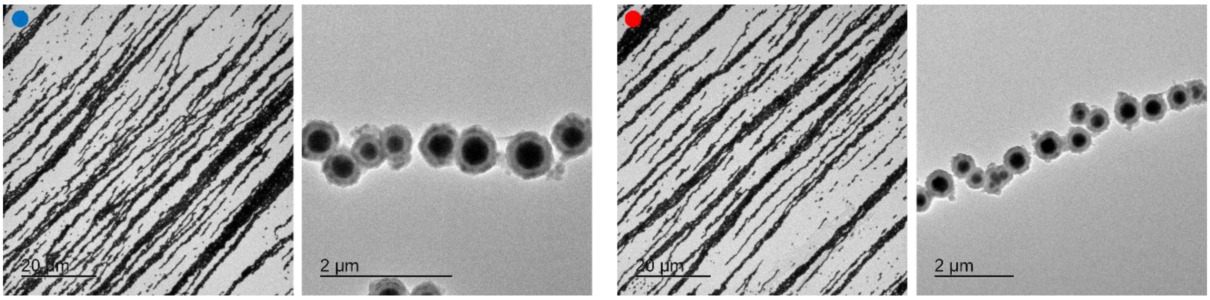

Supplementary Fig.13|TEM images of the LMNP assemblies under the dipolar magnetic field at the center (left) and at the out–of–center (right). The assembled structures of LMNPs show linear chains (anisotropic) regardless of the position, which is significantly different compared with quadrupolar magnetic field assembly.

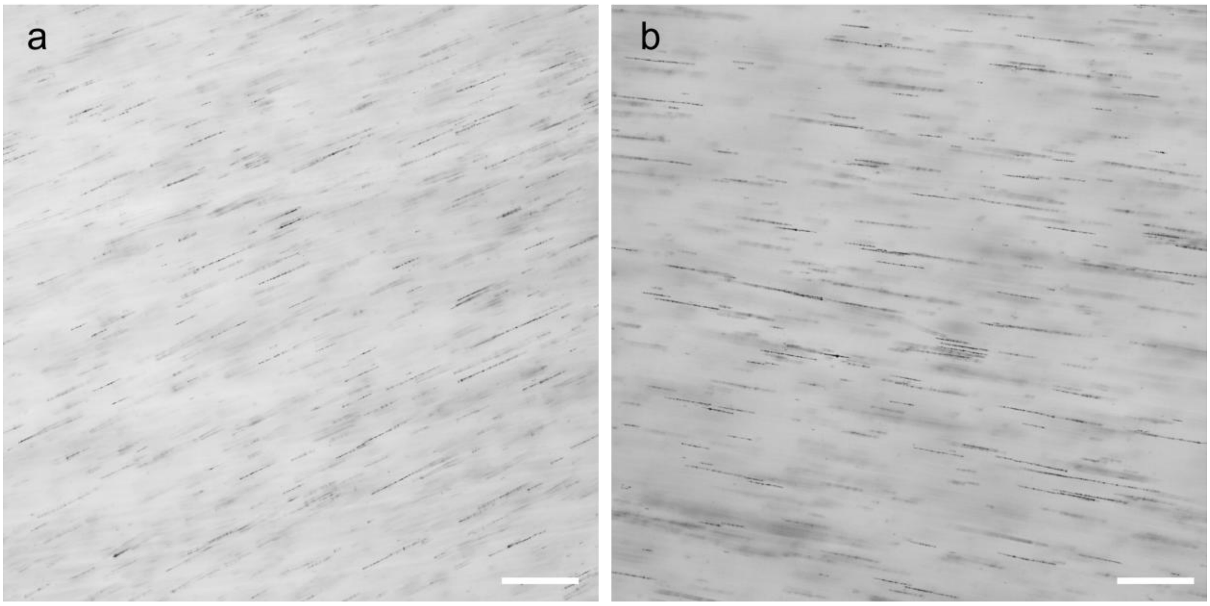

Supplementary Fig.14|Confocal microscope image of assembled nanochain structures (a) before and (b) after tens of twisting cycles. Scale bars are 50 μm.

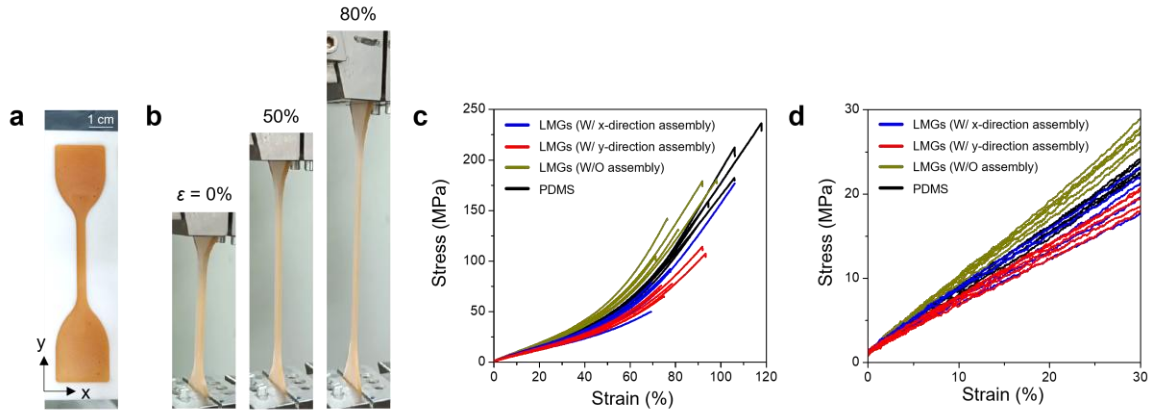

Supplementary Fig.15|Mechanical properties of LMGs. (a) Standard sample for tensile strength test. (b) Photos of LMGs under elongation. (c,d) Strain–stress curve of PDMS and LMGs depending on the assembly direction. (Olive: LMGs cured without external magnetic field, which have random distribution of LMNPs in the elastomeric matrix. Blue and red: LMGs cured under magnetic field along the *x*– and *y*–axis respectively, whose assembly direction is orthogonal or parallel to the tensile strain.) It shows linear elastic properties up to 30% of strain, regardless of the assembly and its direction. Though the existence of nanoparticle assemblies in the elastomeric matrix affects the Young's modulus, the linear elasticity is well preserved, as shown in Supplementary Fig.12c and d. Because the shear modulus (G) is proportional to Young's modulus (E) in PDMS (E ≈ 3G)[7], LMGs also show linear elastic properties in torsional deformation (Supplementary Fig.17).

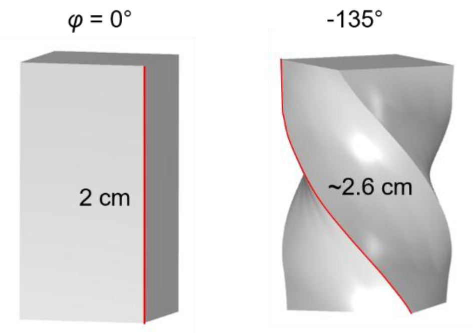

Supplementary Fig.16|Length change of LMG's edge under twisting. When LMG is twisted to 135°, the edge length is elongated around 30% (The edge is marked with a red curve. The shrinkage along the twisting axis is not considered).

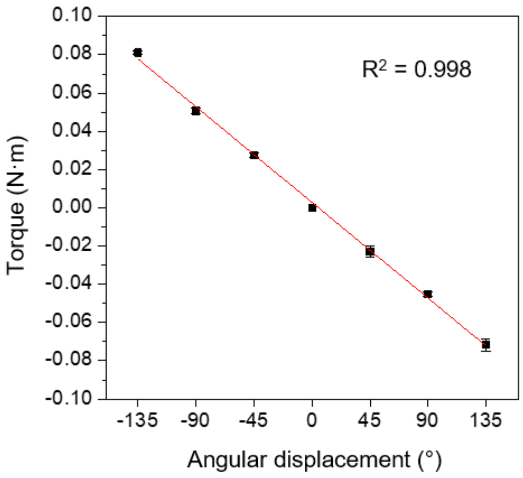

Supplementary Fig.17|Linear correlation between torque and angular displacement. The LMGs show linear elastic property under torsional deformation within a ±135° range of angular displacement.

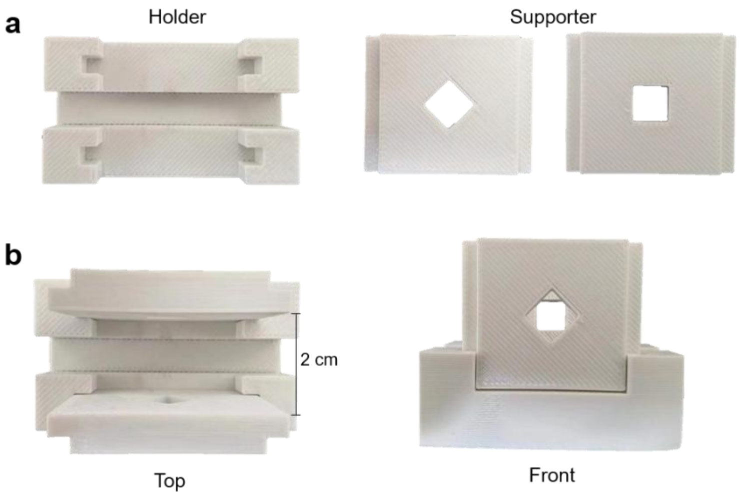

Supplementary Fig.18|3D–printed setups to twist and hold the LMGs. (a) Holder and supporters. (b) Assembled parts.

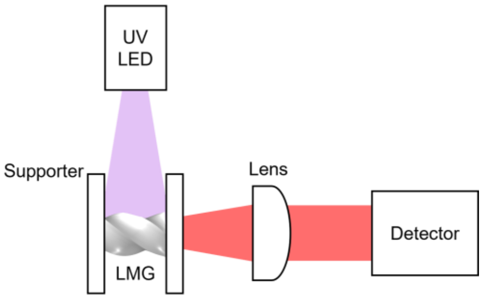

Supplementary Fig.19|Schematic illustration of the optical setup. CPL measurement setup in CPL–200. The UV LED light source is used to evenly excite the overall LMG for CPL measurement. Unless otherwise specified, these experimental configurations were employed for all measurements.

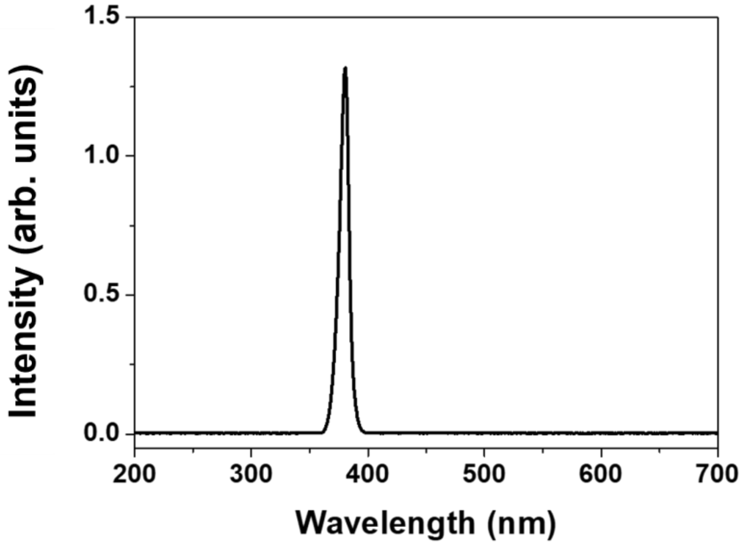

Supplementary Fig.20|Luminescence spectrum of the UV LED used for CPL measurement. The UV LED light source shows a sharp emission peak at 390 nm.

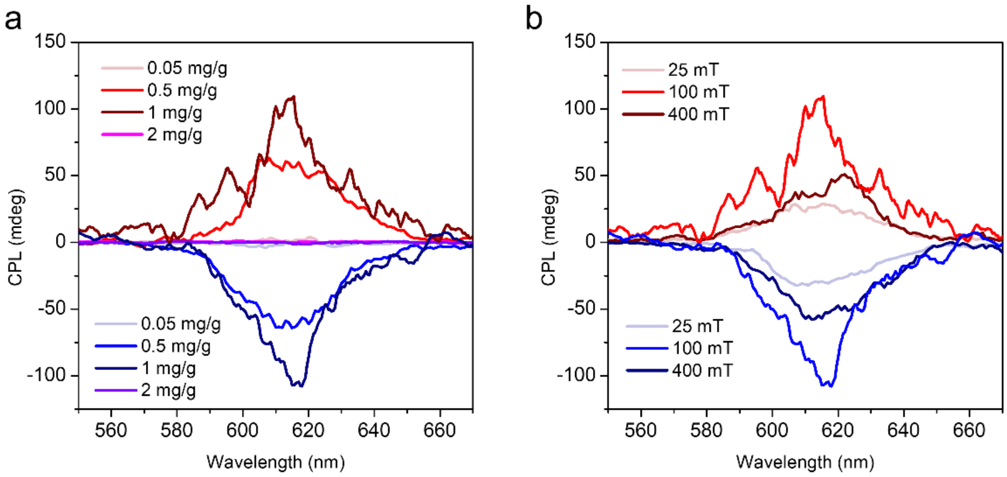

Supplementary Fig.21| CPL spectrum of LMG depending on (a) nanoparticle concentration (nanoparticle mass/gel mass) and (b) magnetic field intensity during assembly (twisting angle φ was set to -135° or 135°).

Increasing nanoparticle concentration from 0.05 mg/g to 1 mg/g significantly enhances CPL intensity up to around 120 mdeg, while future increasing to 2 mg/g will decrease the optical transparency of LMG, leading to decrease in CPL intensity. For magnetic field intensity, since weak intensity (25 mT) cannot guarantee sufficient nanoparticle chain formation, CPL intensity of LMG increases when 100 mT magnetic field intensity was used. However, excessively strong magnetic field (400 mT) reduces CPL intensity due to excessive particle migration toward side of the gel. These results highlight the importance of optimized assembly conditions, including nanoparticle concentration and magnetic field, to obtain optimal CPL performance of LMG.

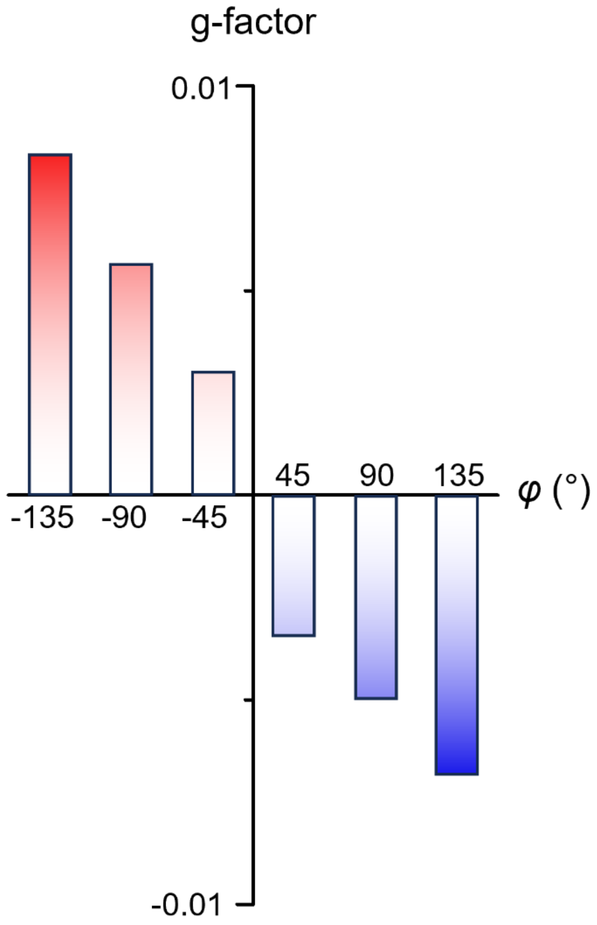

Supplementary Fig.22|Dissymmetry factor (g_{lum}) variation of LMGs depending on twisting angle (φ). g_{lum} of 0.0083, 0.0056, 0.0030, -0.0034, -0.0049, and -0.0068 correspond to φ of -135°, -90°, -45°, 45°, 90°, and 135°. Each g_{lum} is obtained at the peak position.

g_{lum} is calculated by[8]

$$g_{lum} = (\theta/DC) \times (6.98 \times 10^{-5})$$

where:

θ: ellipticity in millidegrees (mdeg)

DC: total luminescence intensity (in volts, V).

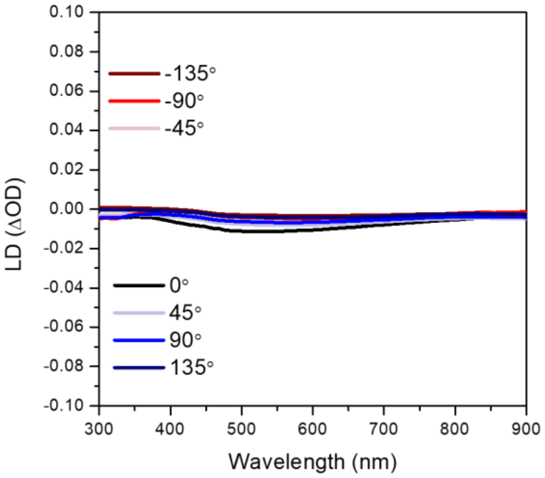

Supplementary Fig.23| LD spectra of LMG depending on φ.

There was a tiny change in LD spectrum under mechanical twisting, however, LD signals were kept in very low level (<0.02) and even decreased when it is twisted.

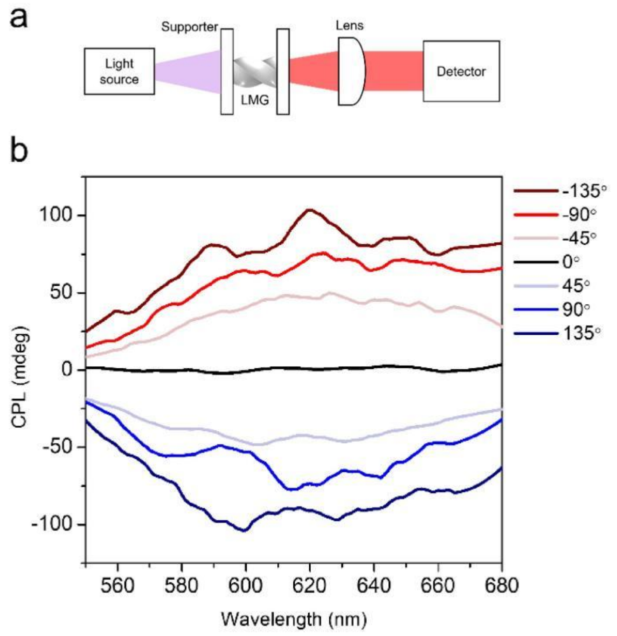

Supplementary Fig.24| (a) Scheme of light path in the measurement of CPL and (b) CPL spectra of LMG depending on φ.

The CPL peak intensity increases with increasing twist angle φ, consistent with the trend observed in the original geometry where the light path is perpendicular to the twisting axis. We note that in the parallel–geometry configuration the detector slit width must be broadened due to reduced transparency, which results in broadened spectral features; however, the qualitative dependence on twist angle remains unchanged. These results confirm that the twisting–induced CPL modulation is an intrinsic property of the twisted LMG and is not an artifact of a specific excitation/collection direction.

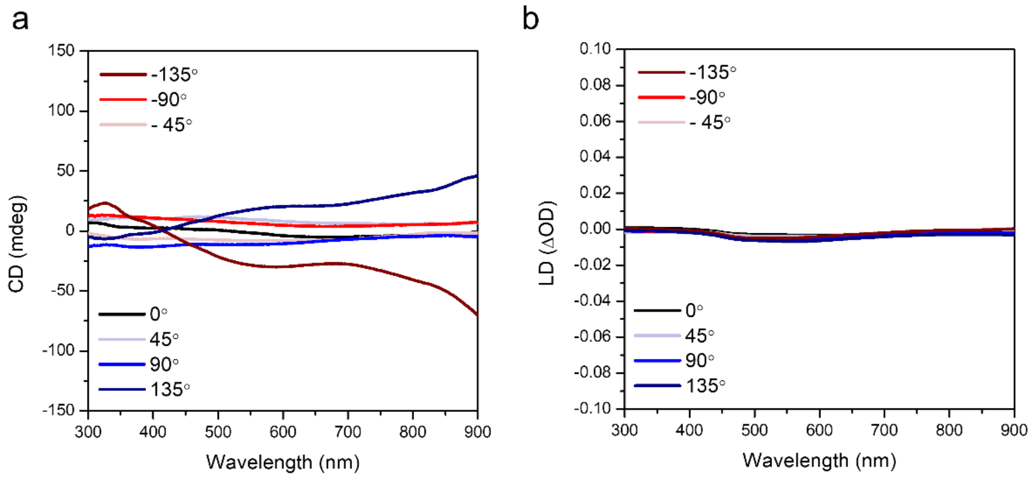

Supplementary Fig.25| CD and LD spectra of bare PDMS depending on *φ*. (a) CD spectra and (b) LD spectra of bare PDMS without nanoparticles.

Though bare PDMS shows CD under twisting, its intensity and angle–dependent change was not sufficient to explain the dynamic CPL properties of LMG. Therefore, the torque–induced reversible CPL properties are responsible for the assembled superstructures in LMG rather than the elastomeric matrix itself.

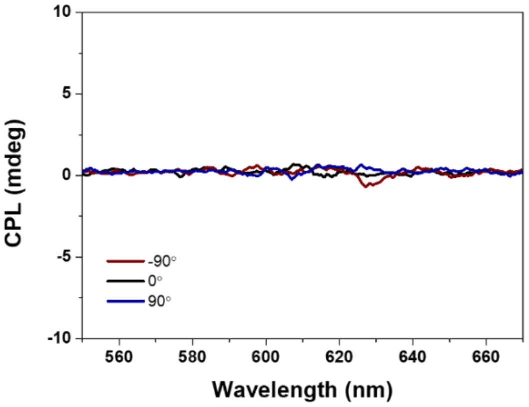

Supplementary Fig.26|CPL spectra depending on the twisting angles of LMGs without magnetic assembly. The random distribution of LMNPs in the elastic matrix shows no CPL peak even though it is twisted, disclosing that liquid crystal–like superstructures are essential for chiroptical properties and deformation of the polymeric matrix does not induce chiroptical properties. Therefore, the measured CPL in LMGs originates from the chiral nematic superstructures of LMNPs in the elastic matrix.

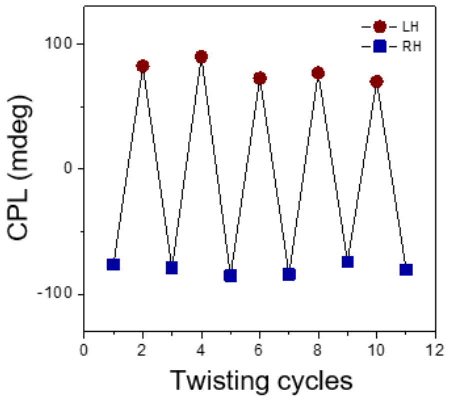

Supplementary Fig.27|Handedness switching property of LMGs one year after fabrication. Due to the protected superstructures of LMNPs by elastic matrix, CPL properties as well as reversible tunability are well maintained.

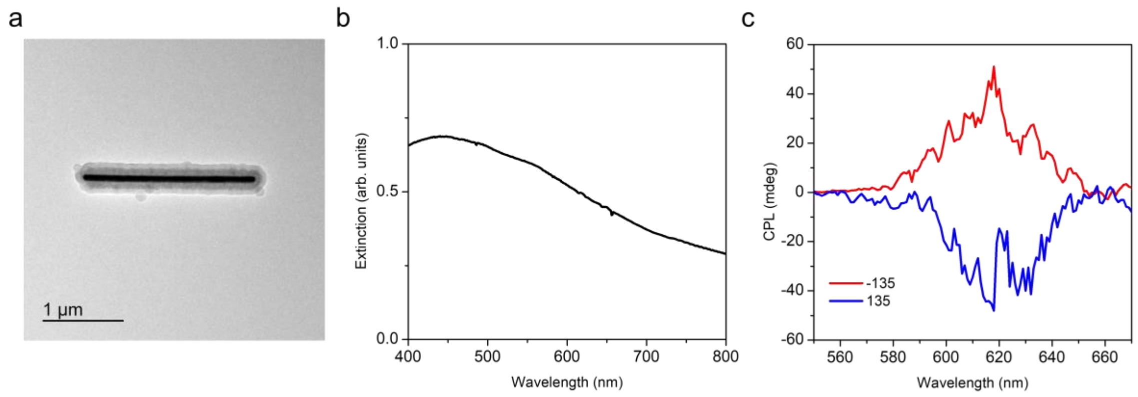

Supplementary Fig.28| (a) TEM image and (b) extinction spectrum of luminomagnetic nanorods, and (c) CPL spectra of LMG made from luminomagnetic nanorods upon twisting (twisting angle φ was set to -135° or 135°).

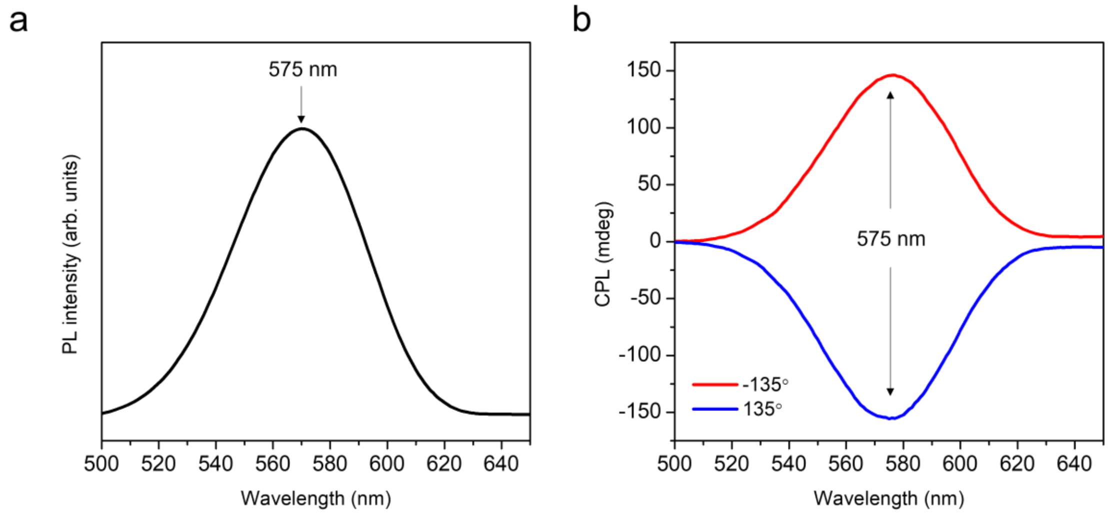

Supplementary Fig.29| (a) PL spectrum of $Tb(DBM)_3(Phen)$ complex and (b) CPL spectra of Tb–LMG under twisting with -135° and 135°.

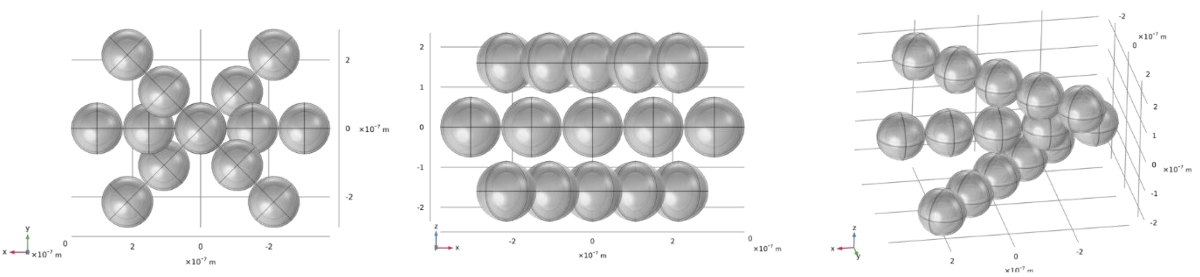

Supplementary Fig.30|Simulation geometry used for optical simulations. The left–handed structures are represented with three nanochains composed of five core–shell NPs. Inter–chain angle is set as 45° between adjacent nanochains.

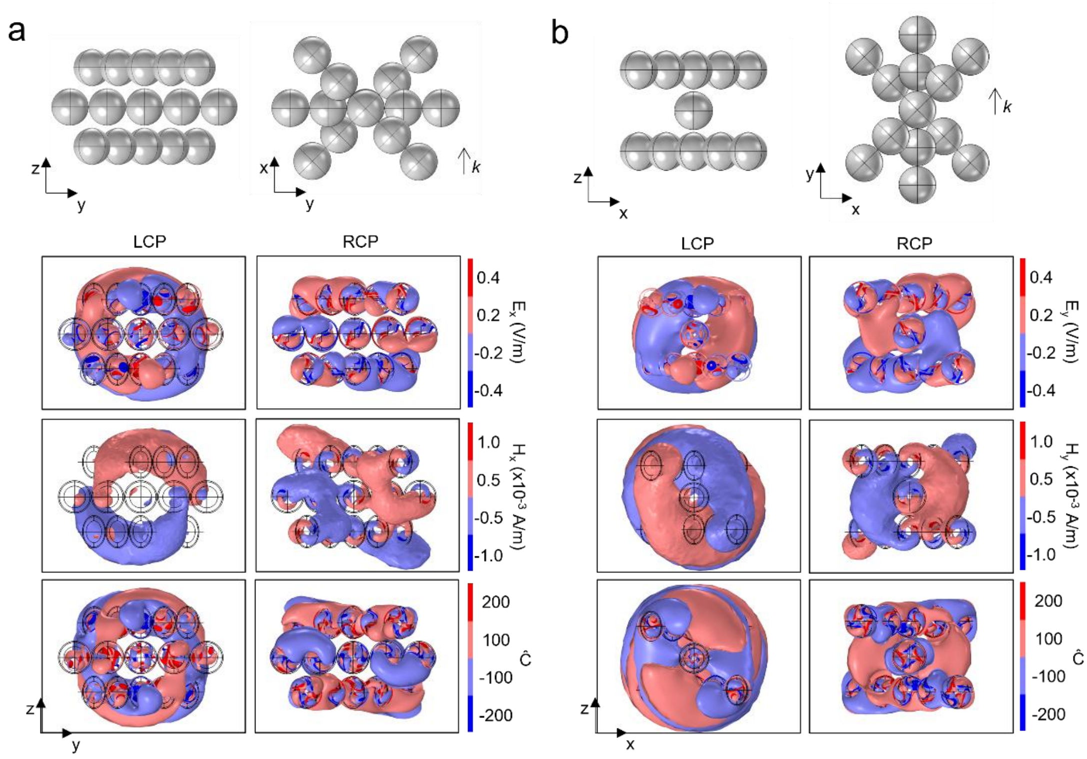

Supplementary Fig.31|Simulation of asymmetric light–matter interactions under varying incident directions. The simulation geometry depicts light incident along the (a) *x*–axis and (b) *y*–axis, both of which are orthogonal to the twisting axis (*z*–axis); the **k**–vector indicates the propagation direction in each case. Spatial distributions are shown for the electric field (**E**), magnetic field (**H**), and superchiral field (**Ĉ**) in the vicinity of the twisted nanochains. All fields were induced by LCP and RCP illumination at a resonance wavelength of 610 nm.

Owing to the intrinsic chirality of the twisted nanochain architectures, the induced electric (**E**), magnetic (**H**), and superchiral (**Ĉ**) field morphologies exhibit a pronounced dissymmetry between LCP and RCP excitation. Notably, this chiroptical response remains consistent regardless of the incident light direction (*x*– or *y*–axis), demonstrating the robustness of the structural chirality (Supplementary Fig.29). Under LCP illumination, the electromagnetic energy is predominantly localized with round morphology around the center. In contrast, RCP excitation results in a more diffuse spatial distribution, where the fields are spread across the nanochains. This helicity–dependent confinement—characterized by central concentration for LCP and peripheral dispersion for RCP—is consistent with the field behavior observed under excitation along the *z*–axis (Fig.5f), further confirming the strong chiroptical coupling of the system.

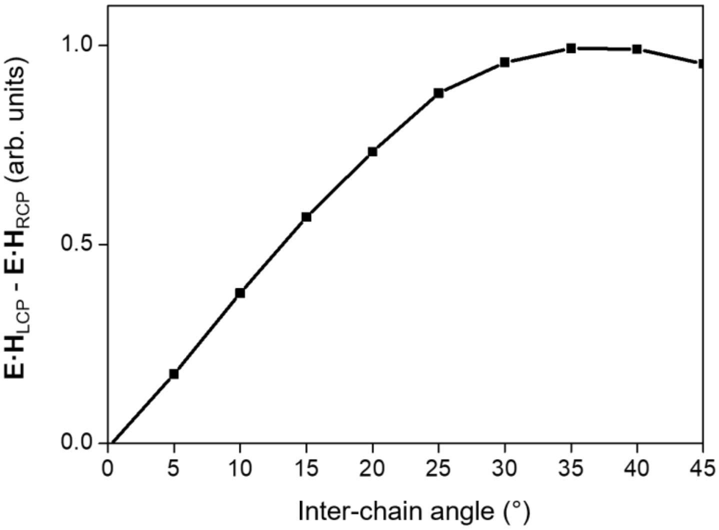

Supplementary Fig.32|The optical asymmetry (E·H$_{LCP}$-E·H$_{RCP}$) in the vicinity of twisted chains, depending on the inter-chain angle. The optical asymmetry increases monotonically until the twist angle reaches 40°.

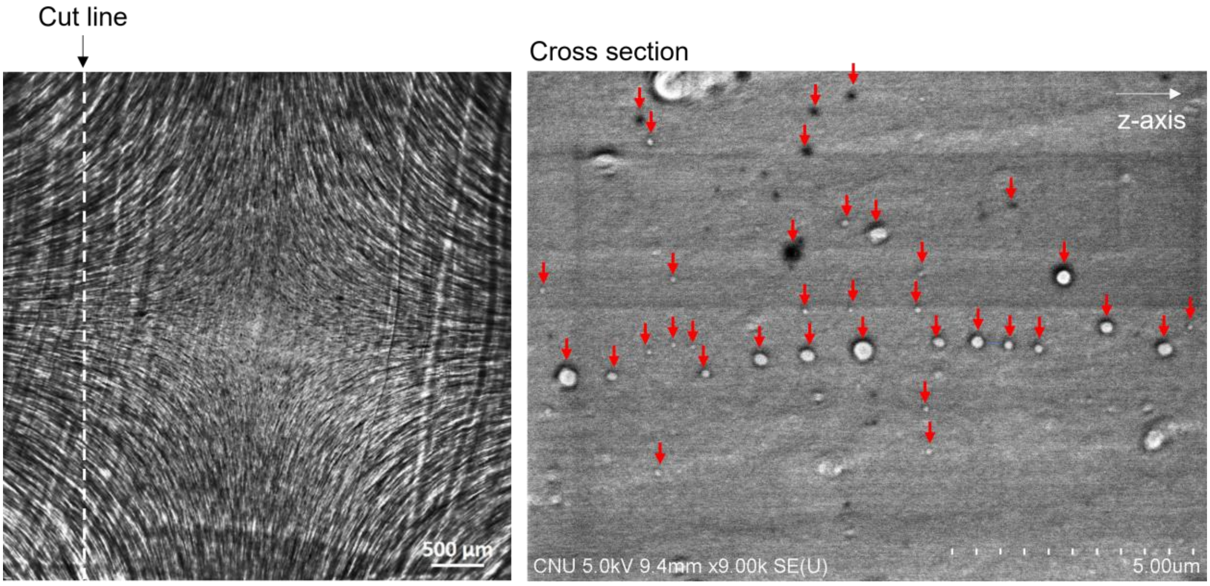

Twisting angle (φ)	Inter-chain angle (average)
45°	0.00088°
90°	0.00176°
135°	0.00264°

Supplementary Fig.33|Quantifying the inter–chain angle of superstructures. Thirty-five chains (red arrows) were observed along the *z*–axis over a 13.40 μm range in the SEM image of the LMG cross–section. Some black regions represent voids where LMNPs were located. The average inter–chain distance is

$$13.40\ \mu m / 34 = 0.39\ \mu m$$

When φ = 45° (45° for 2 cm twist axis), the twist ratio is 22.5° / 10^{-2} m. The average inter–chain angle is

$$(22.5° / 10^{-2}\ m) \times (3.9 \times 10^{-7}\ m) = 0.00088°$$

Therefore, the average inter–chain angles were 0.00088°, 0.00176°, and 0.00264°, corresponding to twisting angles of 45°, 90°, and 135°, respectively.

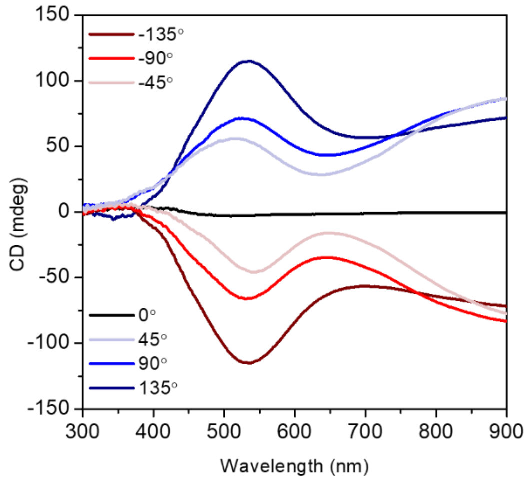

Supplementary Fig.34|CD spectra of LMG under different *φ*.

To experimentally confirm CD in our system, we measured the CD spectra of LMGs at different twisting angles *φ*. The CD response becomes increasingly pronounced as *φ* increases. This observation supports our interpretation that the measured CPL originates from the chiroptical response of the twisted LMG.

Importantly, we note that the measured CD sign is opposite to the measured CPL sign. This result is fully consistent with our interpretation that the observed CPL does not originate from intrinsic chiral emission of the luminophore, but instead arises from a CD–induced optical filtering mechanism. Specifically, when achiral photoluminescence propagates through the twisted LMG (acting as a chiral attenuator), the LCP and RCP components experience different attenuation due to the CD of the medium. Consequently, the transmitted/emerging emission becomes imbalanced between LCP and RCP, yielding an apparent CPL signal. Because CD measures the difference in extinction/absorbance between LCP and RCP, while CPL reflects the resulting difference in transmitted emission intensities after propagation, the CD and CPL can naturally exhibit opposite signs depending on the sign conventions of CD and CPL. Therefore, the observed opposite CD–CPL sign relation provides additional evidence supporting that the measured CPL is governed by the CD response of twisted LMGs.

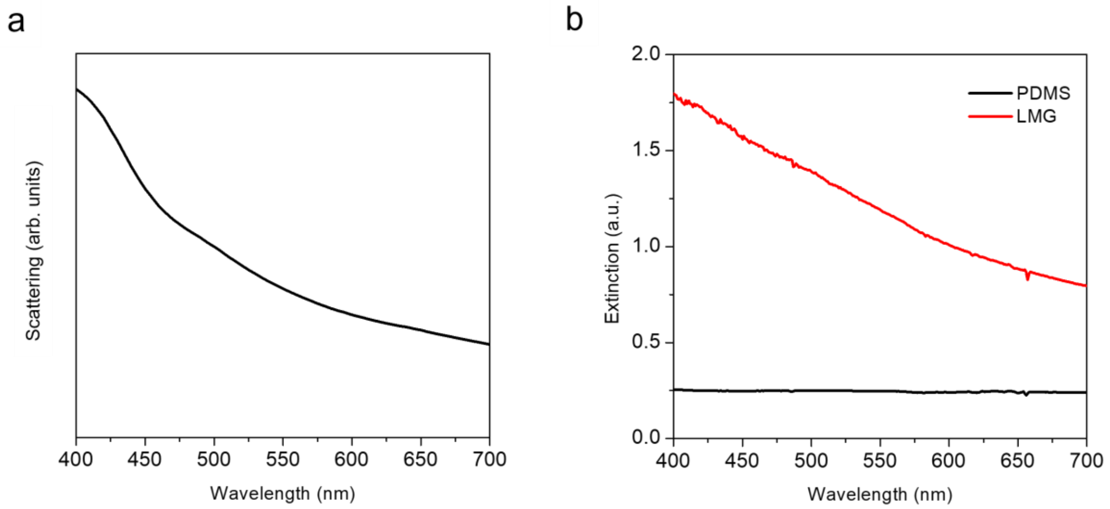

Supplementary Fig.35| (a) Calculated scattering spectrum of twisted nanochain structures. (b) Measured extinction spectrum of PDMS and LMG.

In our twisted LMG, scattering indeed exists because LMNP act as resonant/strongly scattering objects (Supplementary Fig.35a). The extinction spectrum of LMG resembles that of LMNPs (Supplementary Fig.35b).

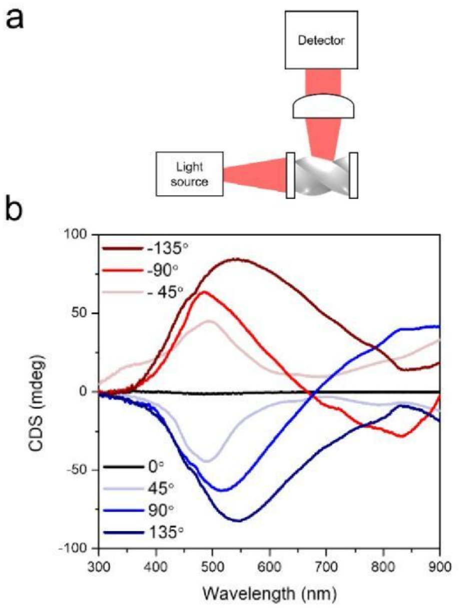

Supplementary Fig.36| (a) Scheme of light path in the measurement of Circular differential scattering (CDS) and (b) CDS spectrum of LMG depending on φ.

To evaluate contribution of scattering–based chirality to the observed CPL experimentally, we measured circular differential scattering (CDS) by adapting the standard CD measurement geometry: instead of detecting extinction along the propagation direction, the detector was positioned orthogonally to the incident beam to selectively collect scattered light (Supplementary Fig.36a). This approach allows us to isolate scattering dissymmetry (CDS) from extinction–based CD. The twisted LMG displays a measurable CDS spectrum; however, the CDS peak exhibits the opposite sign relative to the extinction CD (Supplementary Fig.36b). This observation suggests that the scattering–induced chiral response would oppose, rather than reinforce, the helicity observed in CPL. Accordingly, CDS is unlikely to be the primary origin of the measured CPL, but rather a negative contribution to the measured CPL of the LMG. We therefore attribute the CPL mainly to the intrinsic chiroptical response reflected in the CD signal, while the influence of CDS on CPL is minor under our experimental conditions.

REFERENCES

1. Kim, Y. et al. Reconfigurable chiroptical nanocomposites with chirality transfer from the macro- to the nanoscale. *Nat. Mater.* **15**, 461–468 (2016).
2. Yeom, J. et al. Chiromagnetic nanoparticles and gels. *Science* **359**, 309–314 (2018).
3. Choi, W. J. et al. Terahertz circular dichroism spectroscopy of biomaterials enabled by kirigami polarization modulators. *Nat. Mater.* **18**, 820–826 (2019).
4. Probst, P. T. et al. Mechano-tunable chiral metasurfaces via colloidal assembly. *Nat. Mater.* **20**, 1024–1028 (2021).
5. Kumar, P. et al. Photonically active bowtie nanoassemblies with chirality continuum. *Nature* **615**, 418–424 (2023).
6. Li, Z. et al. A magnetic assembly approach to chiral superstructures. *Science* **380**, 1384–1390 (2023).
7. Lötters, J. C., Olthuis, W., Veltink, P. H. & Bergveld, P. The mechanical properties of the rubber elastic polymer polydimethylsiloxane for sensor applications. *J. Micromech. Microeng.* **7**, 145 (1997).
8. Nagata, Y., Nishikawa, T. & Suginome, M. Chirality-switchable circularly polarized luminescence in solution based on the solvent-dependent helix inversion of poly (quinoxaline-2, 3-diyl) s. *Chem. Commun.* **50**, 9951 (2014).
