# Supplementary material for: Reversible three-dimensional chirality continuum enabled by luminomagnetic superstructure in gel
Source: Nat Commun. 2026 May 11;17:6319. doi: 10.1038/s41467-026-73140-x (PMC13377049; doi:10.1038/s41467-026-73140-x)
Supplement: Supplementary file 2 — Description of Additional Supplementary Files [file 41467_2026_73140_MOESM2_ESM.pdf]

Description of Additional Supplementary Files

Supplementary Video 1. Side-view simulation of the chiral phase transition of luminomagnetic gel.

Supplementary Video 2. Top-view simulation of the chiral phase transition of luminomagnetic gel.

Supplementary Video 3. Simulation of the superchiral field in the vicinity of twisted nanochains in luminomagnetic gel under illumination with left-handed circularly polarized light (LCP) light at the resonance wavelength of 610 nm.

Supplementary Video 4. Simulation of the superchiral field in the vicinity of twisted nanochains in a luminomagnetic gel under illumination with right-handed circularly polarized light (RCP) light at the resonance wavelength of 610 nm.
