# Supplementary material for: Reversible three-dimensional chirality continuum enabled by luminomagnetic superstructure in gel
Source: Nat Commun. 2026 May 11;17:6319. doi: 10.1038/s41467-026-73140-x (PMC13377049; doi:10.1038/s41467-026-73140-x)
Supplement: Supplementary file 7 — Transparent Peer Review file [file 41467_2026_73140_MOESM7_ESM.pdf]

nature portfolio

Peer Review File

Reversible Three-dimensional Chirality Continuum Enabled by Luminomagnetic Superstructure in Gel

Corresponding Author: Professor Zhiyong Tang

This file contains all reviewer reports in order by version, followed by all author rebuttals in order by version.

Version 0:

Reviewer comments:

Reviewer #1

(Remarks to the Author)
In this manuscript, the authors introduce a decoupled design paradigm that integrates magnetic field-directed achiral superstructures of luminomagnetic nanoparticles (LMNPs) with post-curing mechanical manipulation. Under a quadrupolar magnetic field, LMNPs self-assemble into nematic superstructures that are fixed within elastomers to form a luminomagnetic gel (LMG). This work delivers a modular, solid-state platform for polarization engineering and high-precision/broadband chiroptical technologies. Overall, this work is very interesting, clearly structured, and of broad interest to the readership of Nature Communications. Thus, I recommend publishing the manuscript after the authors address the following questions and concerns.

1. For the 3D superstructures in gel, the distribution of the magnetic field in 3D space is highly correlated to the magnetic-driven assembly of magnetic NPs. Thus, it is recommended to have more information on the distribution of the magnetic field in 3D space and how it correlates with the assembly of LMNPs in 3D space. Current results have a limitation in providing a 3D distribution for both.

2. Regarding the linear dichroism (LD) measurements, could the authors clarify under which state these were conducted? Figure 3 suggests the data were collected in the untwisted state. Could you also describe how LD evolves when the LMG is mechanically twisted? Also, LD is sensitive to the relative orientation between the incident light and the assembled structures. Could the author also provide the orientation-dependent LD results for the 3D assembled structure in gel?

3. It would be valuable to understand how employing anisotropic nanostructures (e.g., nanorods or nanoplates) might affect their CPL properties. Additionally, could the authors comment on whether the nanoparticle concentration and the magnetic field intensity during assembly influence the final optical intensity of CPL after the mechanical twisting step?

4. The work utilizes Eu^{3+} ions to achieve excellent luminescence. We are interested in knowing if doping with other rare-earth ions could be a feasible strategy to modulate or tune the emission properties for broader applications.

5. The manuscript demonstrates impressive twisting within a range of -135° to 135° for CPL tuning. Could this torsional range potentially be further increased to achieve an even stronger CPL response, or are there inherent limitations?

6. The speed of optical response is a critical parameter for practical device integration. Would it be possible to measure the response speed of the CPL induced by the twisting mechanism reported in this work? Same for the relaxation responses from chiral states to achiral states during the reversible transition, as shown by the authors.

7. We also noted some minor issues with the reference formatting; for instance, Reference 16 is missing the volume number and page numbers. A thorough check of the reference list would be beneficial.

Reviewer #2

(Remarks to the Author)
This work reports the reversible tuning of chirality continuum by magnetically assembling Eu-doped $Fe_3O_4@SiO_2$ LMNPs

into superstructures in a gel followed by mechanical twisting. Eu-doped Fe3O4@SiO2 LMNPs were prepared as building blocks with magnetic responses and light emission properties. By placing four magnets into a square manner, the authors created a quadrupolar magnetic field with interesting magnetic field distribution. In such a magnetic field, the LMNPs were assembled into linear chains and aligned parallelly to local magnetic fields. The unique symmetry of the quadrupolar magnetic field eliminates the linear dichroism, which was frequently observed in conventional dipolar magnetic field. Upon twisting the superstructures assembled in the quadrupolar magnetic field, the authors demonstrated circularly polarized emission with continuous and reversible over the structural handedness and emission profiles. In addition to experiments, simulation was also used to monitor and probe the magnetic field properties and structure transition during mechanical twisting.
This work introduced a quadrupolar magnetic field to assemble Fe3O4@SiO2 LMNPs, which eliminates the LD effects in superstructures and applied mechanical twisting to create chirality continuum over structural handedness and light emission polarization. Experimental and theoretical consideration were introduced in this work to understand the phase transition and optical properties. A continuous structure transition and chirality continuum were realized in this work.
However, there are a few key points to be clarified in current manuscript. I would expect the authors to address these concerns in a major revision on current manuscript, which is critical to understand the novelty and progress presented in this work, including the structure transition from the initial to the chiral configuration during twisting, the contribution of LB/LD/CD to the observed CD signals, and some clarifications on the results in current manuscript. The detailed comments are listed before for the authors' consideration during revision.

1) Add length scales to Figure 3b.
2) Please explain why the LD is eliminated in the quadrupolar magnetic field because it is important for the reader to understand the novelty of this work.
3) In nematic liquid crystals, molecules have orientational order and no positional order. In the nematic domain, all the molecules are expected to be aligned unidirectionally. In figure 3e, however, the magnetic nanoparticles were assembled into complex superstructures, with changing chain directions and four-fold rotation symmetry. Please clarify the superstructures formed under the quadrupolar magnetic field.
4) Since the starting superstructures are nonlinear before twisting, it is not clear how the structures shown in Figure 3e evolve into the superstructures in Figure 4b. Confocal microscope image after twisting might be useful to understand the twisted structures. If possible, monitoring superstructure changes under confocal microscope by gradually increasing the twisting angles is important to understand the phase transition.
5) Some experimental details were missing in current manuscript, including how to measure the LD spectra.
6) The LD spectra of the gel after twisting should be measured to eliminate the contribution of LD to the observed CD signals. Although LD spectra were provided for superstructures before twisting, LD after twisting might be needed after twisting because of the changes in the superstructures.
7) To demonstrate the structure uniformity in Z-axis, confocal z-stacking is expected to provide detailed structural information before and after twisting.
8) The observed CD is a sum contribution of LD, linear birefringence, and CD. To claim that the observed CD is due to structural chirality, the LD and LB contribution should be eliminated. Therefore, the contribution of LB, LD, and CD to the CD signals after mechanical twisting should be carefully studied.

Reviewer #3

(Remarks to the Author)
This study used a quadrupolar magnetic field to self-assemble luminomagnetic nanoparticles (LMNPs) in a higher-order array and cured within a polydimethylsiloxane (PDMS) elastomer. LMNPs were synthesized by coating a Eu-containing shell on the surface of Fe3O4/silica core-shell nanoparticles. The fabricated luminomagnetic gel (LMG) exhibited elastic behavior under mechanical strain and presented twisted alignment of the LMNPs by application of left or right-handed twists. This resulted in the appearance of elastic and reversible handedness for the CPL, demonstrating the advantage of embedding within the elastomer. The fabrication method used in this study is intriguing, and the elastic changes of the CPL yielded interesting results. However, the mechanism of CPL induction in LMG remains unclear, and other parts still need further improvements. Therefore, this work needs to be reconsidered after addressing following comments.

1. Origin of CPL in the twisted LMG was not clearly explained. Were the simulation results in Fig 5f obtained from LCP and RCP illuminations? Why circularly polarized lights were utilized for these simulations? The CPL measurements must be obtained from non-circular polarized light. And which direction circularly polarized lights were incident from? Are they any changes in the E, H, and superchiral field depending on the incident direction? Typically, chiral nanostructures present such directional dependency. Elaborate this part and supplement descriptions on the simulation methods.

2. I also have a same question on the experimental CPL data. It is presumed that CPL responses from the twisted LMG vary from the direction of the UV illumination and the detector direction. It is necessary to obtain the CPL responses from other directional illumination different from the set-up of Fig S15 for clearer understanding of the CPL results.

3. Does LMG present circular dichroism?

4. The size of LMNP is about 300 nm. This is large enough to scatter lights in the LMG. How does it affect to CD and CPL in the LMG?

5. Under mechanical strains, PDMS experiences alignments of polymeric chains resulting in large birefringence. This can significantly affect to overall chiroptical reseponses. For instance, the Figure S11b shows stress-whitening of the samples,

that indicate the optical responses already include birefringence from the polymeric chain alignments. Were there any experimental CPL data that could exclude the effects of such birefringence during the twisting?

6. Twisting can induce the breakage of the nanoparticle chain alignments beside the helical alignments. The degree of breakage might become greater in greater degree of twist. This may destroy helical shaped alignments? Are there any experimental data?

Version 1:

Reviewer comments:

Reviewer #1

(Remarks to the Author)
The manuscript has been significantly improved after the authors carefully addressed all the reviewers' question and concerns. Thus, i recommend the acceptance of this work to be published in Nature Communications.

Reviewer #2

(Remarks to the Author)
I thank the authors for considering my comments and providing the revised manuscript. All my concerns have been addressed properly and the revised manuscript is sutiable for publication.

Reviewer #3

(Remarks to the Author)
The authors have satisfactorily addressed all of the reviewers' comments. I appreciate the additional experiments and the thorough explanations provided in the Response letter. As a suggestion, if feasible, the authors may consider including some of the key points from the discussion made on Reviewer 3, Comments 2~5 (Figures R3-2 to R3-6), at least in the Supporting Information. This would provide readers with a more comprehensive understanding of the CD and scattering effects in the mechanically strained systems and would be valuable to researchers in the related field.

Open Access This Peer Review File is licensed under a Creative Commons Attribution 4.0 International License, which permits use, sharing, adaptation, distribution and reproduction in any medium or format, as long as you give appropriate credit to the original author(s) and the source, provide a link to the Creative Commons license, and indicate if changes were made.
In cases where reviewers are anonymous, credit should be given to 'Anonymous Referee' and the source.
The images or other third party material in this Peer Review File are included in the article’s Creative Commons license, unless indicated otherwise in a credit line to the material. If material is not included in the article’s Creative Commons license and your intended use is not permitted by statutory regulation or exceeds the permitted use, you will need to obtain permission directly from the copyright holder.

To view a copy of this license, visit https://creativecommons.org/licenses/by/4.0/

For Reviewer #1:

General Comment: In this manuscript, the authors introduce a decoupled design paradigm that integrates magnetic field–directed achiral superstructures of luminomagnetic nanoparticles (LMNPs) with post–curing mechanical manipulation. Under a quadrupolar magnetic field, LMNPs self–assemble into nematic superstructures that are fixed within elastomers to form a luminomagnetic gel (LMG). This work delivers a modular, solid–state platform for polarization engineering and high–precision/broadband chiroptical technologies. Overall, this work is very interesting, clearly structured, and of broad interest to the readership of Nature Communications. Thus, I recommend publishing the manuscript after the authors address the following questions and concerns.

Our response: We sincerely thank the reviewer for the positive and encouraging assessment of our work. We are grateful that the reviewer found the manuscript interesting, clearly structured, and relevant to the broad readership of *Nature Communications*. We also appreciate the constructive questions and concerns raised. In response, we have carefully revised the manuscript, added the necessary explanations, and supplemented the simulations and experimental details where needed. All the modifications are highlighted in the revised manuscript. We believe that these revisions have strengthened the clarity and rigor of the work, and we respectfully hope that the revised manuscript satisfactorily addresses all of the reviewer's comments.

Comment 1: For the 3D superstructures in gel, the distribution of the magnetic field in 3D space is highly correlated to the magnetic–driven assembly of magnetic NPs. Thus, it is recommended to have more information on the distribution of the magnetic field in 3D space and how it correlates with the assembly of LMNPs in 3D space. Current results have a limitation in providing a 3D distribution for both.

Our response: We sincerely thank the reviewer for this important and insightful comment regarding the 3D distribution of the magnetic field and its correlation with the 3D assembly of LMNPs inside the gel. We fully agree that our original manuscript did not sufficiently convey the 3D characteristics of the magnetic field nor explicitly connect them to the 3D superstructure formation.

To address this question, we have revised the manuscript and added new analyses as follows:

The 3D magnetic field distribution and magnetic flux are simulated and presented in below **Figure R1–1**, which incorporates multiple views to clearly illustrate the magnetic field. The side–view analysis demonstrates that the magnetic flux remains orthogonal to the z–axis throughout the sample volume, while the field geometry is invariant along the z direction (**Figure R1–1c**, **1–1e**). Consequently, all x–y planes experience identical magnetic–field

patterns, indicating excellent field uniformity along the z–axis.

Since LMNP nanochains assemble along local magnetic flux lines, the resulting superstructures closely replicate the quadrupolar field geometry. This is demonstrated by the confocal microscope image (Fig. 3e in the revised manuscript), which closely resembles the simulated magnetic flux distribution (top–view in **Figure R1–1b**). These results explicitly establish the direct correspondence between the 3D magnetic–field distribution and the resulting LMNP superstructures.

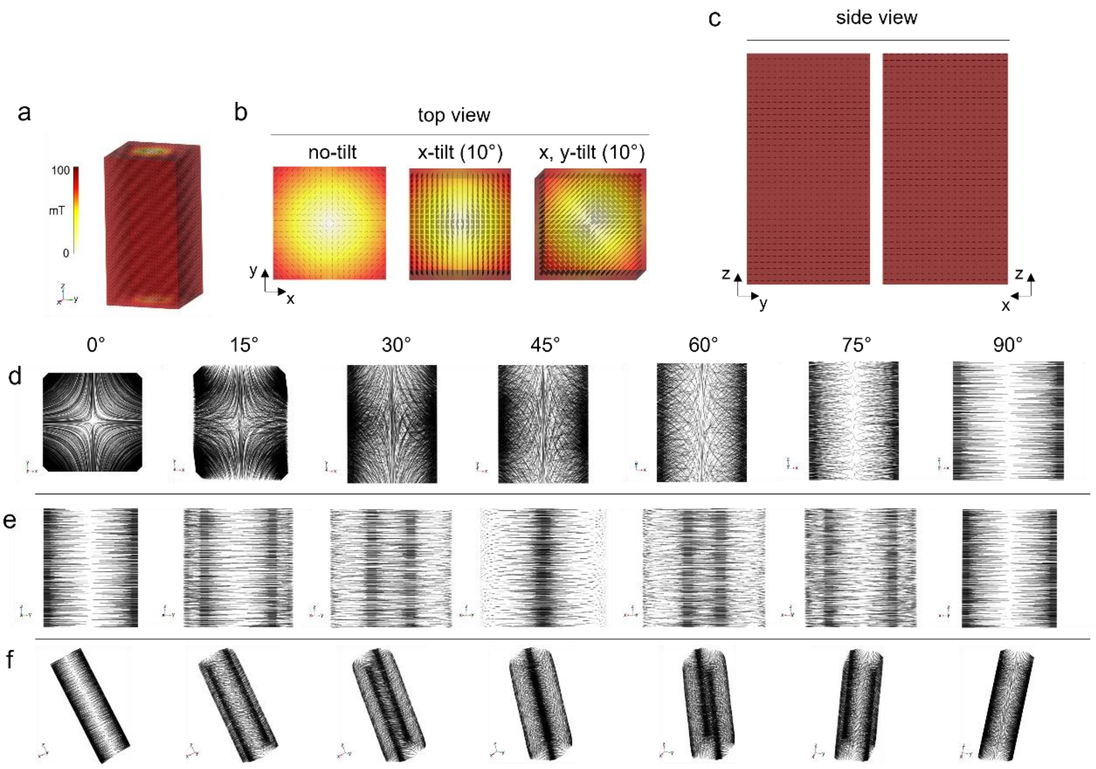

Figure R1–1. Simulated 3D magnetic field distribution within the LMG space under the quadrupolar magnetic–field configuration. (a) Perspective view of the 3D magnetic field distribution within LMG space. (b) Top view of the magnetic field distribution with 0º tilting (no–tilt), 10º tilted view along X–axis (X–tilt) and 10º tilted view along X–axis and Y–axis (X–Y–tilt). (c) Side view of the magnetic field distribution. Magnetic field intensity and direction is presented as color and arrows. (d–f) Visualized magnetic flux images during rotation with different views. (d) Top view of magnetic flux upon rotation with x–axis. (e) Side view of magnetic flux upon rotation with z–axis. (f) Perspective view of magnetic flux upon arbitrary rotation axis.

Our revision: Following the reviewer's insightful suggestion, we have made the following changes in the revised manuscript and supplementary information. Simulated 3D magnetic field distribution within the LMG space under the quadrupolar magnetic–field configuration have been supplemented (please see Supplementary Fig.10 on page S12 in the revised supplementary information; this figure is also presented as **Figure R1–1** in our response), as well as the yellow–highlighted sentences of "To investigate the 3D characteristics of the magnetic field,

we simulated the 3D magnetic field distribution and flux within the LMG space (Supplementary Fig. 10). Side–view analysis demonstrates that the magnetic flux remains orthogonal to the z–axis throughout the sample volume, indicating excellent field uniformity." on page 8, line 13–18 in the revised manuscript.

Comment 2: Regarding the linear dichroism (LD) measurements, could the authors clarify under which state these were conducted? Figure 3 suggests the data were collected in the untwisted state. Could you also describe how LD evolves when the LMG is mechanically twisted? Also, LD is sensitive to the relative orientation between the incident light and the assembled structures. Could the author also provide the orientation–dependent LD results for the 3D assembled structure in gel?

Our response: We sincerely thank the reviewer for these important points regarding the LD measurements. Below is our point-by-point response:

(1) Measurement state of LD:

As correctly noted by the reviewer, the LD spectra shown in Fig. 3 were obtained in the untwisted state. We've clarified this in the revised Figure 3 caption.

(2) LD evolution during twisting:

We have measured LD spectra of LMG under mechanical twisting (**Figure R1–2a and 1–2c**). Although minor spectral variations are observed, the LD signal remains extremely weak and even decrease upon twisting.

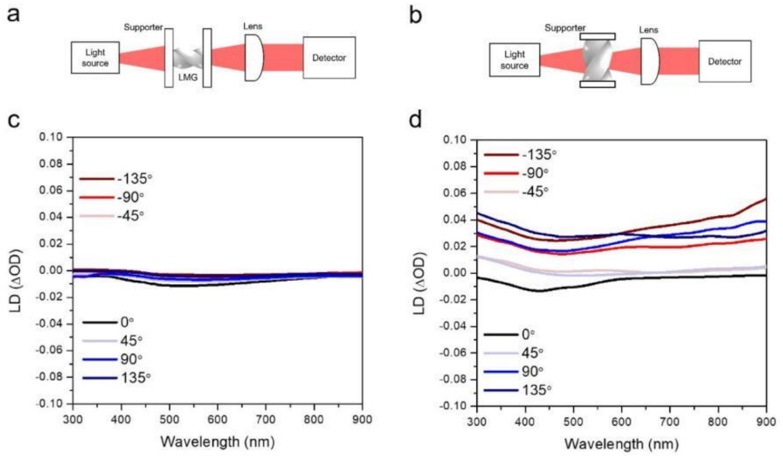

Figure R1–2. Linear dichroism (LD) spectra of LMG depending on twisting angle φ**.** Light paths are varied: (a) light path along twisting axis (z–axis), with light incidents from the x–y plane of LMG; and (b) light path perpendicular to the twisting axis (x– or y–axis), with light incidents from the x–z or y–z plane of LMG. (c,d) Corresponding LD spectra of LMG depending on φ.

(3) Influence of the relative orientation between the incident light and the assembled structures on LD:

We further measured the LD spectra with different light–propagation direction relative to the

twisting axis through rotating the sample by 90º (**Figure R1–2b** and **1–2d**). The higher LD in **Figure R1–2d** than **Figure R1–2c** is due to the preferential alignment of nanochains inside x–y planes along the light path (please refer to **Figure R1–1**). The effective suppression of LD along z-axis of LMG (**Figure R1-2a and R1-2c**) originates from the non-unidirectional geometry of the quadrupolar magnetic field in the x–y plane, which is a key novelty of our approach.

Our revision: Following the reviewer's constructive suggestion, we have added the following results in both the revised manuscript and the revised supplementary information:

(1) We've clarified the LMG state of LD measurement in the revised Figure 3 caption (please see the highlighted part in caption in Fig.3d in the revised manuscript on page 7, line 6).

(2) We've added LD evolution during twisting in the revised supplementary information as Supplementary Fig.23, and corresponding description "In order to determine the possible LD effect, induced by torsion, we characterized the LD under mechanical twisting (Supplementary Fig. 23). Regardless of φ, LD remains at a very low level (<0.02), confirming that LD contributions are negligible and do not influence the observed chiroptical responses." has been added in the revised manuscript on page 10, line 9–12.

Comment 3: It would be valuable to understand how employing anisotropic nanostructures (e.g., nanorods or nanoplates) might affect their CPL properties. Additionally, could the authors comment on whether the nanoparticle concentration and the magnetic field intensity during assembly influence the final optical intensity of CPL after the mechanical twisting step?

Our response: We thank the reviewer for the insightful comments regarding the roles of nanoparticle anisotropy, concentration, and magnetic field intensity in modulating LMG CPL performance. Below is our point-by-point response:

(1) Anisotropic building blocks:

Regarding nanoparticle anisotropy, we chose magnetic nanorod to evaluate its influence on CPL modulation. Magnetic Fe_3O_4 nanorods were successfully synthesized, coated with SiO_2 shell and Eu^{3+} to form Eu–doped Fe_3O_4@SiO_2 luminomagnetic nanorods, and incorporated into LMGs.

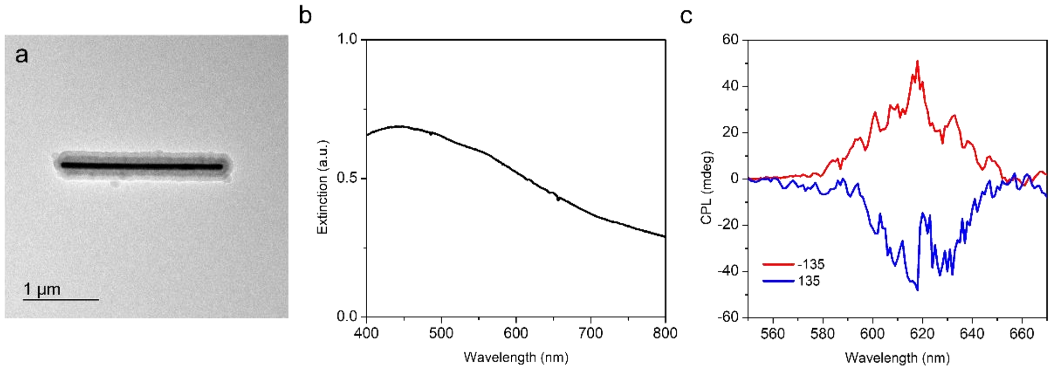

Figure R1–3. (a) TEM image and (b) extinction spectrum of luminomagnetic nanorods, and (c) CPL spectra of LMG made from luminomagnetic nanorods upon twisting (twisting angle φ was set to -135° or 135°).

LMGs fabricated from luminomagnetic nanorods also exhibit twist–induced CPL, confirming the generality of our strategy (**Figure R1–3**). The weaker CPL intensity of nanorod based LMG, compared to spherical–particle–based LMGs at the same particle mass concentration, is likely attributed to the lower number density of the heavier nanorods.

(2) Nanoparticle concentration and magnetic field intensity effect:

We also systematically investigated the effects of nanoparticle concentration and magnetic–field intensity during assembly (**Figure R1–4**). Increasing nanoparticle concentration from 0.05 mg/g to 1 mg/g significantly enhances CPL intensity up to around 120 mdeg. However, a further increase to 2 mg/g reduces the CPL signal due to decreased optical transparency of the LMG. For magnetic field intensity, since weak intensity (25 mT) cannot guarantee sufficient nanoparticle chain formation, CPL intensity of LMG increases when 100 mT magnetic field intensity was used. However, excessively strong magnetic field (400 mT) reduces CPL intensity due to excessive particle migration toward side of the gel. These results highlight the importance of optimized assembly conditions, including nanoparticle concentration and magnetic field, to obtain optimal CPL performance of LMG.

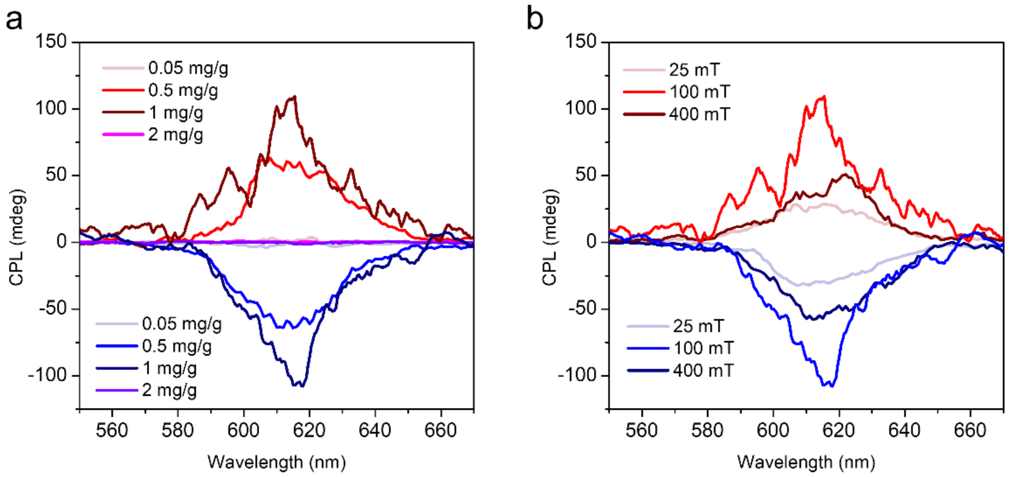

Figure R1–4. CPL spectrum of LMG depending on (a) nanoparticle concentration (nanoparticle mass/gel mass) and (b) magnetic field intensity during assembly (twisting angle φ was set to -135° or 135°).

Our revision: Following the reviewer's constructive suggestion, we have added the following results in both the revised manuscript and the revised supplementary information:

(1) **Figure R1–3** has been added in revised supplementary information as Supplementary Fig.26. The corresponding description "The generality of our strategy was evaluated using luminomagnetic nanorods, which also exhibit twist–induced CPL (Supplementary Fig. 26)."

has been added and highlighted in the revised manuscript on page 10, line 19–20.

(2) **Figure R1–4** has been added in revised supplementary information as Supplementary Fig.21. The corresponding description "In addition, we systematically investigated the effects of nanoparticle concentration and magnetic–field intensity during assembly (Supplementary Fig. 21). Optimization of these parameters is crucial for optimal CPL performance" has been added and highlighted in the revised manuscript spanning from page 9, line22 to page 10, line 3.

Comment 4: The work utilizes Eu^{3+} ions to achieve excellent luminescence. We are interested in knowing if doping with other rare–earth ions could be a feasible strategy to modulate or tune the emission properties for broader applications.

Our response: We thank the reviewer for this valuable suggestion. Doping with other rare–earth ions is indeed a feasible and effective strategy to modulate and tune the emission properties for broader applications. Rare–earth ions such as Tb^{3+}, Sm^{3+}, and Dy^{3+} possess characteristic 4f–4f transitions that enable emissions across a wide spectral range, from visible to near–infrared, thereby offering flexibility in emission color and functionality (*Acc. Chem. Res*. **53**, 2692–2704 (2020)). The LMG developed here is a type of platform to convert mechanical stimuli to chiroptical signal. Therefore, other luminophores as well as other rare–earth ions can be implemented in this LMG platform to show interconversion between twisting and CPL.

As a demonstration, we prepared a Tb–based LMG, which exhibits green emission (~575 nm) and clear twist–induced CPL (**Figure R1–5**). These results confirm that the LMG platform is not limited to Eu^{3+} or a specific emission wavelength and can be broadly extended to other luminophores.

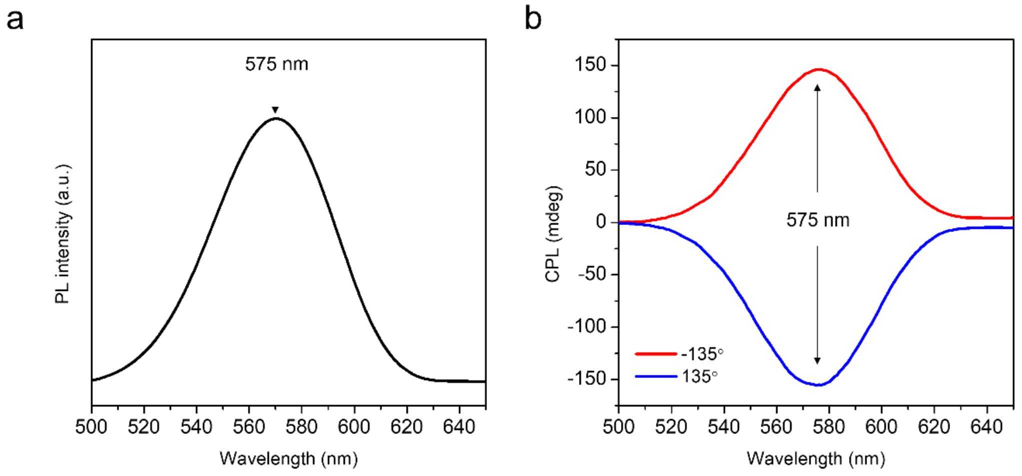

Figure R1–5. (a) PL spectrum of $Tb(dbm)_3Phen$ complex and (b) CPL spectra of Tb–LMG under twisting with -135° and 135°.

Our revision: Following the reviewer's helpful suggestion, we have made the following revisions in both the revised manuscript and the revised supplementary information:

Figure R1–5 has been added in the revised supplementary information as Supplementary Fig.27, and corresponding description "Furthermore, we prepared a Tb–based LMG, which exhibits green emission (~575 nm) and clear twist–induced CPL (Supplementary Fig.27). This confirms that the LMG platform is not limited to nanoparticle geometry or a specific emission wavelength." has been added and highlighted in the revised manuscript on page 10, line 20–23.

Comment 5: The manuscript demonstrates impressive twisting within a range of -135° to 135° for CPL tuning. Could this torsional range potentially be further increased to achieve an even stronger CPL response, or are there inherent limitations?

Our response: We thank the reviewer for this important question. In principle, larger torsional angles can enhance CPL by increasing macroscopic chiral asymmetry. However, the torsional range is limited by the mechanical integrity of the gel. In our PDMS–based system, twisting approaching ~180° leads to mechanical failure and gel broken. We therefore tested twisting angles up to 165°, which indeed produced stronger CPL without compromising structural integrity (**Figure R1–6**).

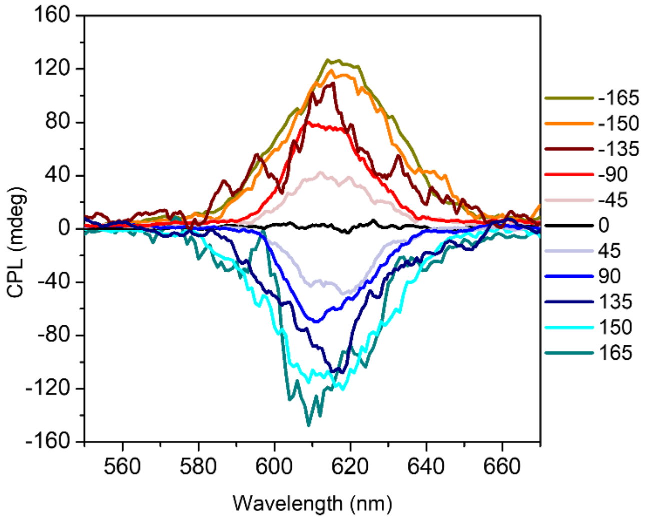

Figure R1–6. CPL spectra of LMG depending on *φ*. The CPL intensity further increases as the *φ* goes beyond ±135°.

Comment 6: The speed of optical response is a critical parameter for practical device integration. Would it be possible to measure the response speed of the CPL induced by the twisting mechanism reported in this work? Same for the relaxation responses from chiral states to achiral states during the reversible transition, as shown by the authors.

Our response: We appreciate the reviewer's insightful comments of the optical response speed, which is critical to application of LMG. The optical response speed is governed by the rate of mechanical twisting. Based on our previous work demonstrating millisecond–scale modulation in colloidal systems (*ACS Nano* **14**, 7152–7160 (2020)), comparable or faster CPL response of LMG is expected with appropriate actuation hardware to twist LMG. Relaxation behavior depends on the elastomer's elasticity when torque is released, whereas enforced reverse twisting can achieve response speeds comparable to forward twisting. Developing a precision mechanical stage capable of twisting maneuvers with target angle in micro–second or milli–second while maintaining optical alignment in CPL spectrophotometer is a significant engineering challenge. Consequently, our current setup would reflect the limitations of the macroscopic instrument rather than the physical limits of the LMG.

Comment 7: We also noted some minor issues with the reference formatting; for instance, Reference 16 is missing the volume number and page numbers. A thorough check of the reference list would be beneficial.

Our response: We thank the reviewer for pointing out the reference formatting issue. We have carefully checked the entire reference list and corrected formatting errors.

Our revision: Following the reviewer's careful comment, we have made the following revisions in "Reference" section in the revised manuscript. Volume number and page numbers are added in reference 6 (6,125–145) and reference 16 (16, 5795–5806). The first letter in journal name is changed to capital letter in reference 17 (Nano) and reference 18 (Nano).

For Reviewer #2:

General Comment: This work reports the reversible tuning of chirality continuum by magnetically assembling Eu–doped Fe3O4@SiO2 LMNPs into superstructures in a gel followed by mechanical twisting. Eu–doped Fe3O4@SiO2 LMNPs were prepared as building blocks with magnetic responses and light emission properties. By placing four magnets into a square manner, the authors created a quadrupolar magnetic field with interesting magnetic field distribution. In such a magnetic field, the LMNPs were assembled into linear chains and aligned parallelly to local magnetic fields. The unique symmetry of the quadrupolar magnetic field eliminates the linear dichroism, which was frequently observed in conventional dipolar magnetic field. Upon twisting the superstructures assembled in the quadrupolar magnetic field, the authors demonstrated circularly polarized emission with continuous and reversible over the structural handedness and emission profiles. In addition to experiments, simulation was also used to monitor and probe the magnetic field properties and structure transition during mechanical twisting.

This work introduced a quadrupolar magnetic field to assemble Fe_3O_4@SiO_2 LMNPs, which eliminates the LD effects in superstructures and applied mechanical twisting to create chirality continuum over structural handedness and light emission polarization. Experimental and theoretical consideration were introduced in this work to understand the phase transition and optical properties. A continuous structure transition and chirality continuum were realized in this work.

However, there are a few key points to be clarified in current manuscript. I would expect the authors to address these concerns in a major revision on current manuscript, which is critical to understand the novelty and progress presented in this work, including the structure transition from the initial to the chiral configuration during twisting, the contribution of LB/LD/CD to the observed CD signals, and some clarifications on the results in current manuscript. The detailed comments are listed before for the authors' consideration during revision.

Our response: We sincerely thank the reviewer for the thoughtful and constructive evaluation of our work. We greatly appreciate the reviewer's clear summary of our approach and for recognizing the novelty of integrating quadrupolar magnetic–field–guided superstructures with mechanically induced chirality continuum.

In response, we have carefully revised the manuscript according to the reviewer's comments and all the modifications are highlighted in the revised manuscript.

Comment 1: Add length scales to Figure 3b.

Our response: We thank the reviewer for careful reading and kind suggestion. We have revised Figure 3b accordingly.

Our revision: Length scale bar has been added to Figure 3b to improve clarity and readability on Page 7 in the revised manuscript, as shown below:

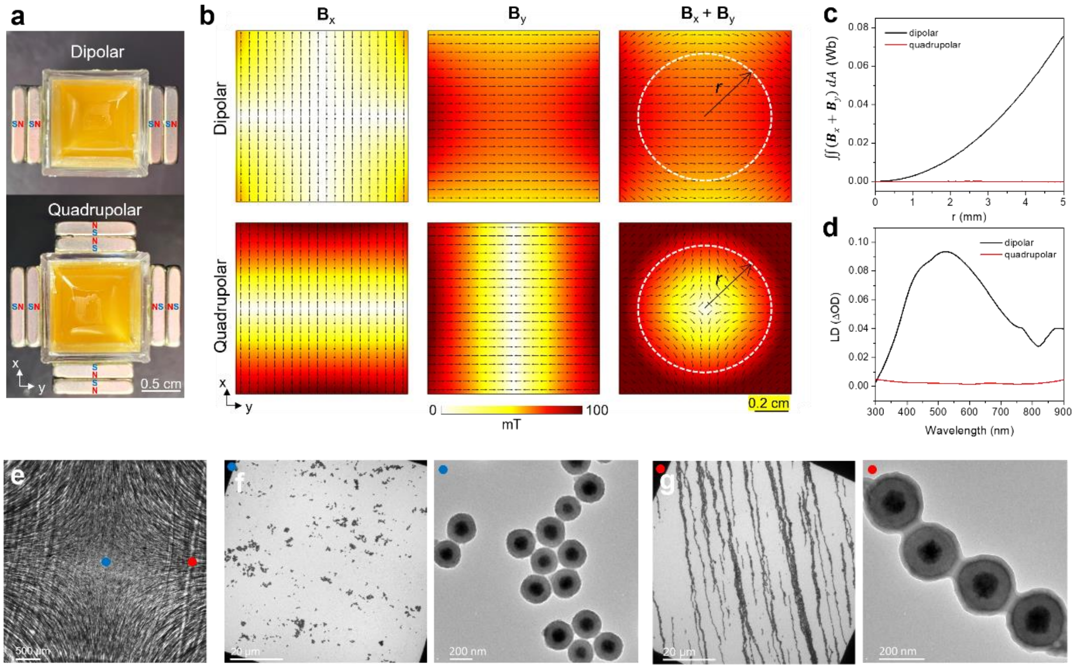

Comment 2: Please explain why the LD is eliminated in the quadrupolar magnetic field because it is important for the reader to understand the novelty of this work.

Our response: We thank the reviewer for this critical question, as it directly relates to the novelty and physical rationale of our approach.

In conventional dipolar magnetic fields, nanochains align along a single dominant axis, producing macroscopic anisotropy and net LD (Figure 3b, 3c and 3d, dipolar magnetic field case, and Supplementary Figure 10). In contrast, the quadrupolar magnetic field is generated by four symmetrically arranged magnets with alternating magnetization directions (Figure 3a). Although local LD may exist microscopically, the global azimuthal symmetry ensures that for the superstructures formed in LMG, LD contributions cancel out macroscopically (Figure 3b and 3c, quadrupolar magnetic field case), resulting in vanishing net LD (Figure 3d). This symmetry–enabled LD elimination is a key novelty of our approach.

Our revision: Following the reviewer's constructive suggestion, we've supplemented the discussion of magnetic field difference between dipolar and quadrupolar magnet arrays, and the reason for LD elimination in quadrupolar case as follows: "Therefore, the non–unidirectional geometry of the quadrupolar magnetic field can effectively eliminate the formation of unidirectional superstructure, which is the origin of strong LD in the dipolar magnet array directed system." on page 8, line 8–11, in the revised manuscript (modified part highlighted in the revised manuscript).

Comment 3: In nematic liquid crystals, molecules have orientational order and no positional order. In the nematic domain, all the molecules are expected to be aligned unidirectionally. In figure 3e, however, the magnetic nanoparticles were assembled into complex superstructures, with changing chain directions and four–fold rotation symmetry. Please clarify the superstructures formed under the quadrupolar magnetic field.

Our response: We thank the reviewer for pointing this out. The assemblies formed under the quadrupolar magnetic field indeed differ from conventional unidirectional nematic phases. In quadrupolar magnetic field, the magnetic dipole–dipole interaction aligns LMNPs into nanochain structures in local area (orientational order, no positional order). When considering in large area, the quadrupolar magnetic field provides global four–fold symmetry structures (no orientational order, positional order) in LMG systems. Therefore, this novel quadrupolar magnetic field arrangement enables local orientational order like nematic phase and produce global positional order, which can remove LD.

Because the quadrupolar magnetic field guides the LMNPs along the magnetic flux, the assembled superstructures (Fig. 3e) resemble the quadrupolar magnetic flux (black arrows in Fig. 3b, B_x+B_y). To clarify this, we further simulated detailed 3D magnetic field geometry in quadrupolar magnet arrays (below **Figure R2–1**). The superstructures can be engraved in the LMG guided by quadrupolar magnetic field geometry, as evidenced by the identical structures observed in Figure 3e and **Figure R2–1b** (top view first image). Therefore, 3D superstructure configuration follows the magnetic field geometry as below, exhibiting quadrupolar symmetry in the x–y plane, and displaying unidirectional pattern in x–z and y–z planes.

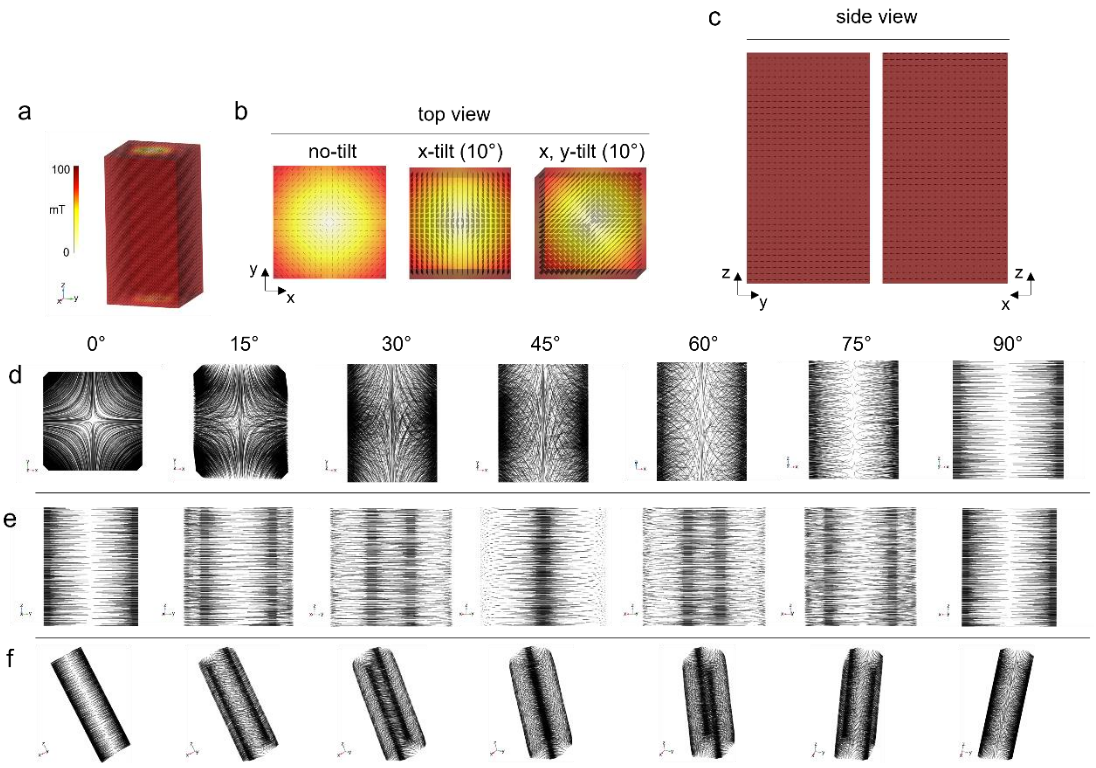

Figure R2–1. Simulated 3D magnetic field distribution within the LMG space under the quadrupolar magnetic–field configuration. (a) Perspective view of the 3D magnetic field distribution within LMG space. (b) Top view of the magnetic field distribution with 0º tilting (no–tilt), 10º tilted view along X–axis (X–tilt) and 10º tilted view along X–axis and Y–axis (X–Y–tilt). (c) Side view of the magnetic field distribution. Magnetic field intensity and direction is presented as color and arrows. (d–f) Visualized magnetic flux images during rotation with different views. (d) Top view of magnetic flux upon rotation with x–axis. (e) Side view of magnetic flux upon rotation with z–axis. (f) Perspective view of magnetic flux upon arbitrary rotation axis.

Our revision: Following the reviewer's constructive suggestion, we have made the following revisions in both the revised manuscript and the revised supplementary information:
Figure R2–1 has been added in the revised supplementary information as Supplementary Fig.10, and corresponding description "To investigate the 3D characteristics of the magnetic field, we simulated the 3D magnetic field distribution and flux within the LMG space (Supplementary Fig. 10). Side–view analysis demonstrates that the magnetic flux remains orthogonal to the z–axis throughout the sample volume, indicating excellent field uniformity" has been added and highlighted on page 8, line 13–18, in the revised manuscript.

Comment 4: Since the starting superstructures are nonlinear before twisting, it is not clear how the structures shown in Figure 3e evolve into the superstructures in Figure 4b. Confocal microscope image after twisting might be useful to understand the twisted structures. If possible, monitoring superstructure changes under confocal microscope by gradually increasing the twisting angles is important to understand the phase transition.
Our response: We thank the reviewer for raising this important point. We agree that clarifying the structural evolution during twisting is essential.
First, we would like to clarify that Fig. 3e and Fig. 4b visualize different aspects and dimension of the LMG, and therefore they should not be interpreted as a direct one–to–one morphological transformation. Specifically, Fig. 3e is a confocal microscope image acquired from a thin, sliced LMG specimen (x–y plane) and highlights the LMNP superstructures generated under the quadrupolar magnetic field, showing features consistent with the field pattern (B_x + B_y, Fig. 3b). In contrast, Fig. 4b (top view) is a CT image of the full bulk LMG, which captures the macroscopic torsional deformation of the entire body after twisting. In the CT reconstruction, the top surface appears larger than the bottom surface due to view setting (shown in the revised **Figure R2–2**). Therefore, the apparent discrepancy between Fig. 3e and Fig. 4b mainly originates from differences in sample dimensions (thin slice vs bulk body) and imaging modalities (confocal vs CT), rather than an abrupt change of the superstructure morphology.
To address the reviewer's kind suggestion of confocal imaging after twisting, we note that performing confocal imaging on a twisted LMG is experimentally challenging in our current

setup. Confocal observation requires a thin, flat specimen (at most a few millimeters in thickness) placed stably on the objective lens, while twisting requires applying controlled torsion with mechanical fixtures. Due to the limited space between the objective lens and the sample stage, as well as the lack of a compatible torsional stage that can maintain the sample under deformation without introducing motion artifacts, it is not feasible to twist a thin LMG slice during confocal imaging. For this reason, we employed CT imaging to characterize the structure of the twisted bulk LMG.

Importantly, the CT cross–sectional (x–y plane) images indicate that the global boundary and shape of the x–y plane remain largely unchanged before and after twisting (**Figure R2–3**), suggesting that twisting mainly introduces angular displacement about the z–axis rather than compressive/distortive deformation in the x–y plane. Since the initially assembled achiral LMNP superstructures are embedded and fixed within the elastomeric matrix, they deform continuously together with the matrix during twisting. Thus, the "phase transition" from achiral to chiral superstructures can be understood as a continuous, torsion–driven reorganization at the macroscopic level, where the superstructures preserve their local integrity in the x–y plane while acquiring a z–axis angular bias through interlayer rotational displacement along the sample thickness. In other words, twisting creates a progressive inter–chain angular offset along the z–direction, which converts the non–chiral architecture into a macroscopically chiral superstructure.

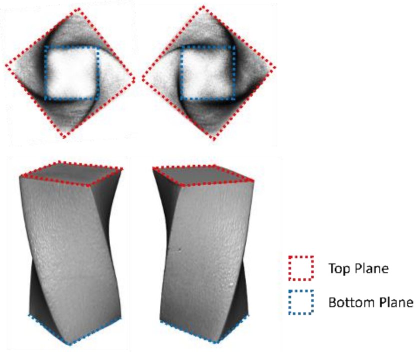

Figure R2–2. **CT images of LMG under twisting**. The edges of top and bottom plane are marked as red– and blue–dotted box respectively.

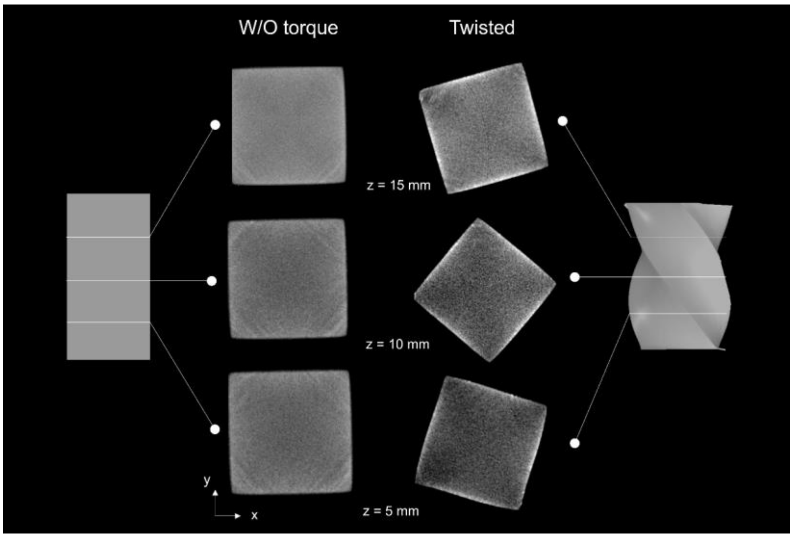

Figure R2–3. **CT cross section (x–y plane) images of LMG with different z–positions.** The z–position of each CT cross section images is marked at the corresponding LMG scheme.

Our revision: Following the reviewer's valuable suggestion, we have revised the manuscript by replacing the original Figure 4b with Figure R2–2. This updated figure is now included on page 9 of the revised manuscript, as shown below:

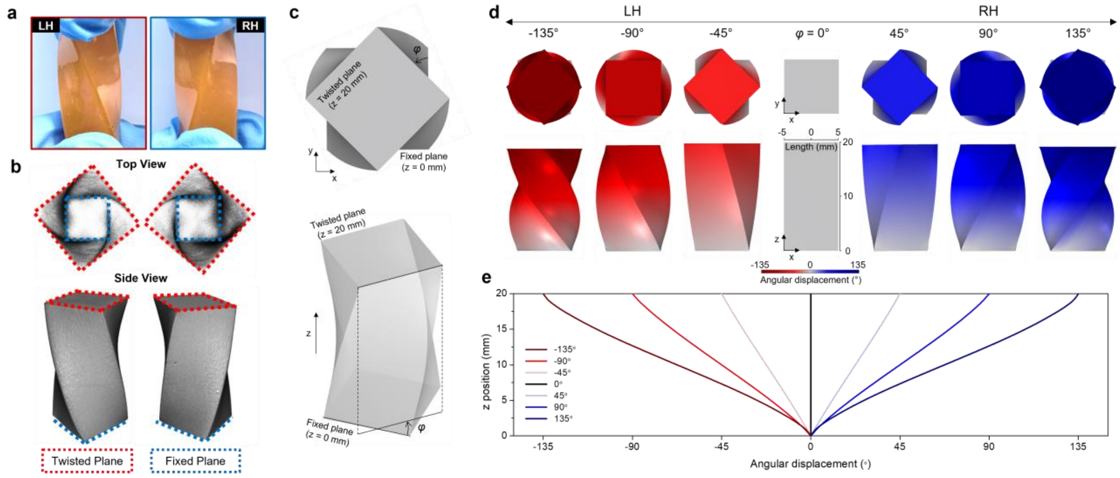

Comment 5: Some experimental details were missing in current manuscript, including how to measure the LD spectra.

Our response: We sincerely thank the reviewer for carefully identifying this important omission. We fully agree that the experimental description of how the linear dichroism (LD) spectra were measured was insufficiently detailed in the original manuscript. Following is our response:

The CD and LD of LMG were measured with CD spectrophotometer (J-1500, Jasco Inc., Japan) in the set up with light source, LMG, and detector aligned in a row (**Figure R2–4**), which is normal light path in CD spectrophotometer.

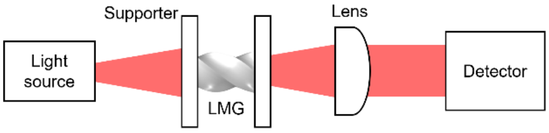

Figure R2–4. Schematic illustration of measurement of linear dichroism (LD) and circular dichroism (CD).

Our revision: Following the reviewer's important comment, we have provided LD measurement scheme as Supplementary Fig.9 (corresponding to Figure R2-4) and corresponding description "The CD and LD were measured in ordinary CD spectrophotometer (J-1500, Jasco Inc., Japan) setup whose light source, sample, and detector is in a row. Unless otherwise specified, these experimental configurations were employed for all measurements." in the revised supplementary information.

Comment 6: The LD spectra of the gel after twisting should be measured to eliminate the contribution of LD to the observed CD signals. Although LD spectra were provided for superstructures before twisting, LD after twisting might be needed after twisting because of the changes in the superstructures.

Our response: We thank the reviewer again for prompting these clarifications. We have measured the LD spectra of the gel after twisting (**Figure R2–5**), which could improve the completeness and interpretability of our LD analysis.

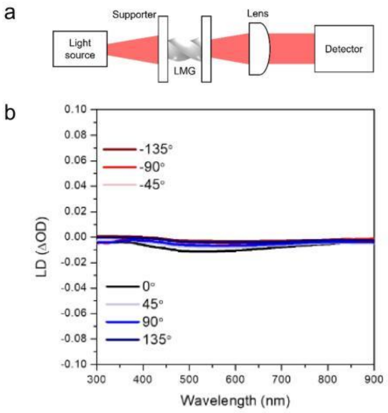

Figure R2–5. (a) Scheme of light path in the measurement of LD and (b) LD spectra of LMG depending on φ.

There are tiny changes in LD spectrum up twisting. However, LD signals were kept in very low level and even decreased when it is twisted. Therefore, we can conclude that the LD effect on CD signals is negligible.

Our revision: According to reviewer's insightful comment, we have added LD spectra under twisting (Figure R2–5) in the revised supplementary information as Supplementary Fig. 23, and

corresponding description “In order to determine the possible LD effect, induced by torsion, we characterized the LD under mechanical twisting (Supplementary Fig.23). Regardless of φ, LD remains at a very low level (< 0.02), confirming that LD contributions are negligible and do not influence the observed chiroptical responses” has been added and highlighted on page 10, line 9–12 in the revised manuscript.

Comment 7: To demonstrate the structure uniformity in Z–axis, confocal z–stacking is expected to provide detailed structural information before and after twisting.

Our response: We thank the reviewer for this helpful suggestion regarding the three–dimensional structural uniformity of the superstructures.

In order to demonstrate the uniformity of the assembled superstructures, we provide the following data. First, from the simulation data, we know that the magnetic field geometry and strength are identical regardless of z–position in the sample positioned region where assembly occurs (**Figure R2–1**). Second, in the CT data, shading patterns are identical in different z–position before and after twisting (**Figure R2–3**). Third, in the confocal microscope images, all nanochains alignment remains the same across different z–positions at the same x–y position (**Figure R2–6**). These results collectively demonstrate that the LMG inner structures are uniform along the z-axis. Regarding the structure after twisting, while confocal imaging of twisted samples is experimentally not feasible as noted in our response to Comment 4, the consistent and reversible CPL response upon twisting (Fig. 4) suggests that the structural uniformity along the z-axis is preserved during LMG deformation.

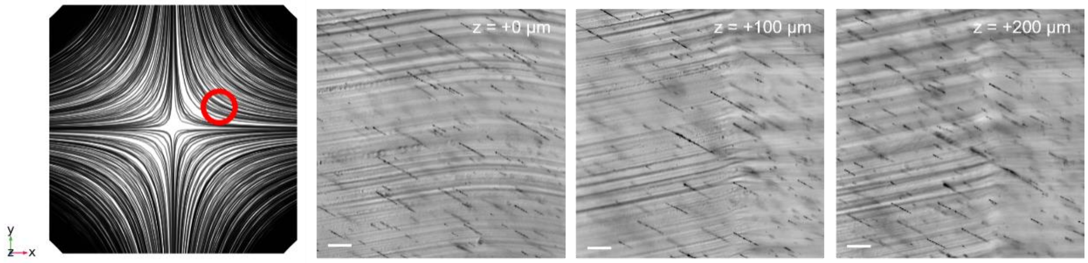

Figure R2–6. Confocal microscope images with different z–position. Scale bars are 20 μm. Red circle in first scheme shows the x–y position where the confocal microscope image is taken.

Our revision: Following the reviewer’s constructive suggestion, we have added confocal z–stacking images (Figure R2-6) in the revised supplementary information as Supplementary Fig.11, and corresponding description “This is further supported by confocal z–stack images showing identical alignment in different x–y planes (Supplementary Fig.11)” has been added and highlighted on page 8, line 17–18, in the revised manuscript.

Comment 8: The observed CD is a sum contribution of LD, linear birefringence, and CD. To

claim that the observed CD is due to structural chirality, the LD and LB contribution should be eliminated. Therefore, the contribution of LB, LD, and CD to the CD signals after mechanical twisting should be carefully studied.

Our response: We appreciate the reviewer's important comment. As the reviewer correctly pointed out, the observed CD is a sum contribution of LD, LB, and CD.

To address this, we first directly measured the LD of our LMG system under various twisting angles (**Figure R2–5**). The results show that the LD signal remains consistently low (<0.02) across all tested twist angles. This indicates that linear anisotropy in absorption is negligible in our system. Regarding LB, we note that LD and LB are intrinsically related through the Kramers-Kronig transformation—both originate from structural anisotropy, and a low LD implies a correspondingly low LB in the absence of strong anomalous dispersion (*J. Synchrotron Rad.* **5**, 995–997 (1998); *J. Phys. Chem. B* **103**, 6371–6383 (1999)). This is also supported by the four-fold symmetry of our superstructures (as discussed in response to Comment 3), which inherently suppresses macroscopic anisotropy and thus minimizes any potential LB contribution.

Given the very low LD values and the symmetry-enabled suppression of anisotropy, we conclude that the contributions of both LD and LB to the observed CD signals are negligible. Therefore, the CD response we observe primarily originates from the true structural chirality of the superstructures assembled in LMGs.

For Reviewer #3:

General comment: This study used a quadrupolar magnetic field to self–assemble luminomagnetic nanoparticles (LMNPs) in a higher–order array and cured within a polydimethylsiloxane (PDMS) elastomer. LMNPs were synthesized by coating a Eu–containing shell on the surface of Fe3O4/silica core–shell nanoparticles. The fabricated luminomagnetic gel (LMG) exhibited elastic behavior under mechanical strain and presented twisted alignment of the LMNPs by application of left or right–handed twists. This resulted in the appearance of elastic and reversible handedness for the CPL, demonstrating the advantage of embedding within the elastomer. The fabrication method used in this study is intriguing, and the elastic changes of the CPL yielded interesting results. However, the mechanism of CPL induction in LMG remains unclear, and other parts still need further improvements. Therefore, this work needs to be reconsidered after addressing following comments.

Our response: We sincerely thank the reviewer for recognizing the novelty of our fabrication strategy and the elastic, reversible CPL behavior enabled by the LMG platform. We also appreciate the reviewer's insightful and constructive comments, particularly regarding the mechanism of CPL induction. These suggestions encouraged us to more rigorously investigate the influence of the elastomeric matrix on the chiroptical properties of the system.

In response, we have substantially revised the manuscript to address all points raised. All the modifications are highlighted in the revised manuscript to ensure clarity. We hope the revised manuscript satisfactorily addresses the concerns and believe the work is now significantly strengthened.

Comment 1: Origin of CPL in the twisted LMG was not clearly explained. Were the simulation results in Fig 5f obtained from LCP and RCP illuminations? Why circularly polarized lights were utilized for these simulations? The CPL measurements must be obtained from non–circular polarized light. And which direction circularly polarized lights were incident from? Are they any changes in the E, H, and superchiral field depending on the incident direction? Typically, chiral nanostructures present such directional dependency. Elaborate this part and supplement descriptions on the simulation methods.

Our response: We sincerely thank the reviewer for this highly insightful and technically important comment regarding the physical origin of the measured CPL in the twisted luminomagnetic gel (LMG), and the rationale for using circularly polarized illumination in our simulations.

First, we would like to clarify that the luminophore embedded in the LMNPs is intrinsically achiral, and thus its intrinsic emission does not produce CPL. Therefore, the experimentally observed CPL signal—measured under non–circularly polarized excitation—does not originate

from the luminophore itself, but instead arises from the chiral optical response of the twisted LMG, which is formed by the twisted LMNP superstructures.

In our system, the twisted LMG acts as a macroscopically chiral and anisotropic optical medium, which exhibits a different optical response to LCP and RCP components. As a result, after luminescence is generated, the emitted photons propagate through the twisted LMG and experience differential extinction of LCP and RCP components. This polarization–dependent propagation effectively converts initially non–chiral luminescence into an apparent CPL signal (i.e., CD–induced CPL). In other words, the observed CPL is governed by the chiroptical asymmetry of the twisted LMG rather than by intrinsic chiral emission of the luminophore.

Accordingly, the simulation in Fig. 5f was performed using LCP and RCP incident light, because the simulation was designed to quantify the intrinsic chiral optical asymmetry of the twisted LMNP superstructures (i.e., their differential interaction with LCP vs RCP), which is the fundamental origin of the CD–induced CPL observed experimentally. We have now clarified in the revised manuscript that LCP/RCP simulations are used to evaluate the chiroptical response of the twisted structure, rather than to mimic the excitation polarization used in CPL experiments.

Regarding the illumination direction, the circularly polarized light in the simulation was incident along the +z direction, which corresponds to the twisting axis of the LMNP superstructure. We chose this direction to ensure physical consistency with the experimental geometry where the detected CPL originates from emission propagating through the twisted structure along the axis of interest. We agree that chiral nanostructures could exhibit direction–dependent chiroptical responses and have done the simulation under varying incident directions (below **Figure R3–1**). We have also added a clearer description of the coordinate system, incidence direction, polarization definition, and superchiral–field calculation in the revised Methods.

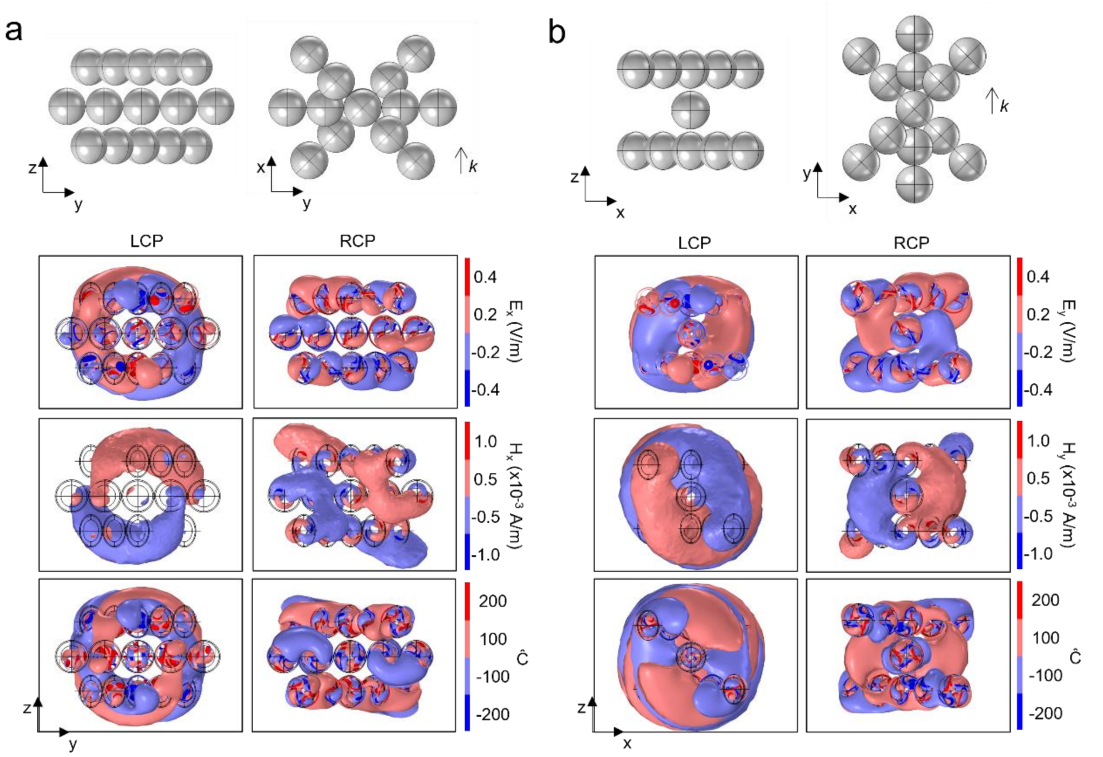

Figure R3–1. Simulation of asymmetric light–matter interactions under varying incident directions. The simulation geometry depicts light incident along the (a) x–axis and (b) y–axis, both of which are orthogonal to the twisting axis (z–axis); the k–vector indicates the propagation direction in each case. Spatial distributions are shown for the electric field (E), magnetic field (H), and superchiral field (Ĉ) in the vicinity of the twisted nanochains. All fields were induced by LCP and RCP illumination at a resonance wavelength of 610 nm.

Our revision: Following the reviewer's constructive suggestion, we have made the following revisions in both the revised manuscript and the revised supplementary information:

(1) We've clarified the incident light direction of the optical simulation in the revised Figure 5 caption (please see the highlighted part in caption in Fig.5f in the revised manuscript on page 11, line 8-9).

(2) We've modified discussion about mechanism of CPL induction by adding description "to evaluate its optical response to left- and right-handed circularly polarized light (LCP and RCP)" in the revised manuscript on page 11, line 11–12, adding description "differs more significantly than" in the revised manuscript on page 11, line 17, adding description "surrounding the twisted nanochains exhibits a pronounced asymmetry between LCP and RCP incidence. The twisted LMG acts as a macroscopically chiral and anisotropic optical medium with distinct responses to LCP and RCP components. As a result, achiral luminescence propagating through it undergoes differential extinction of its LCP and RCP components, converting the emission into an apparent CPL signal." in the revised manuscript on page 11, line 18–20 and page 12, line 1–3; adding description "of the superchiral field" in the revised manuscript on page 12, line 3.

(3) We have added simulation results with different light incident directions in Supplementary Fig. 29 (corresponding to Figure R3–1) and corresponding description "Owing to the intrinsic chirality of the twisted nanochain architectures, the induced electric (E), magnetic (H), and superchiral (Ĉ) field morphologies exhibit a pronounced dissymmetry between LCP and RCP illumination. Notably, this chiroptical response remains consistent regardless of the incident light direction (x– or y–axis), demonstrating the robustness of the structural chirality (Supplementary Fig. 29). Under LCP illumination, the electromagnetic energy is predominantly localized with round morphology around the center. In contrast, RCP excitation results in a more diffuse spatial distribution, where the fields are spread across the nanochains. This helicity–dependent confinement—characterized by central concentration for LCP and peripheral dispersion for RCP—is consistent with the field behavior observed under excitation along the z–axis (Fig.5f), further confirming the strong chiroptical coupling of the system." in the revised supplementary information.

(4) We have also supplemented the simulation method as follows: "Circularly polarized light was modeled by the superposition of two orthogonal linearly polarized waves–x- and y-components–with a ±λ/4 phase shift applied to the y-component (for incident light along the z-axis). Simulation space was surrounded by perfectly matched layer (perfect absorbing boundary conditions). The superchiral fields (Ĉ) were calculated using the following expression:

$$C = -\frac{\omega\varepsilon_0}{2} Im[\boldsymbol{E}^* \cdot \boldsymbol{H}]$$

where E and H represent the complex electric and magnetic field vectors, respectively, ω is the angular frequency of the incident light, and $\varepsilon 0$ is the vacuum permittivity. To quantify the chiral enhancement, the local superchiral field was normalized to that of a circularly polarized plane wave in free space (C_0). Regions where the normalized enhancement factor $|\hat{C}| = |C/C_0| > 1$ indicate the generation of superchiral light of the twisted nanochain structures." on page 14, line 20–30 in the revised manuscript.

Comment 2: I also have a same question on the experimental CPL data. It is presumed that CPL responses from the twisted LMG vary from the direction of the UV illumination and the detector direction. It is necessary to obtain the CPL responses from other directional illumination different from the set–up of Fig S15 for clearer understanding of the CPL results.

Our response: We thank the reviewer for this important suggestion. The current measurement setup was designed to detect the light which is on the twisting axis (z–axis). And this direction is on our purpose that can effectively reduce linear dichroism due to four–fold symmetry of superstructures assembled under quadrupolar magnetic field. But we agree that because the twisted LMG is a macroscopically chiral and anisotropic object, the observed CPL may depend on the illumination direction, collection direction, and relative orientation between the sample,

excitation beam, and detector.

To address this point, we performed additional CPL measurements under an alternative optical geometry in which the light path is parallel to the twisting axis, different from the configuration used in Fig. S15. The new results (**Figure R3–2**) show that the twist–angle–dependent CPL behavior is preserved: the CPL peak intensity around ~610 nm increases with increasing twist angle ϕ, consistent with the trend observed in the original geometry where the light path is perpendicular to the twisting axis. We note that in the parallel–geometry configuration the detector slit width must be broadened due to reduced transparency, which results in broadened spectral features; however, the qualitative dependence on twist angle remains unchanged.

These results confirm that the twisting–induced CPL modulation is an intrinsic property of the twisted LMG and is not an artifact of a specific excitation/collection direction.

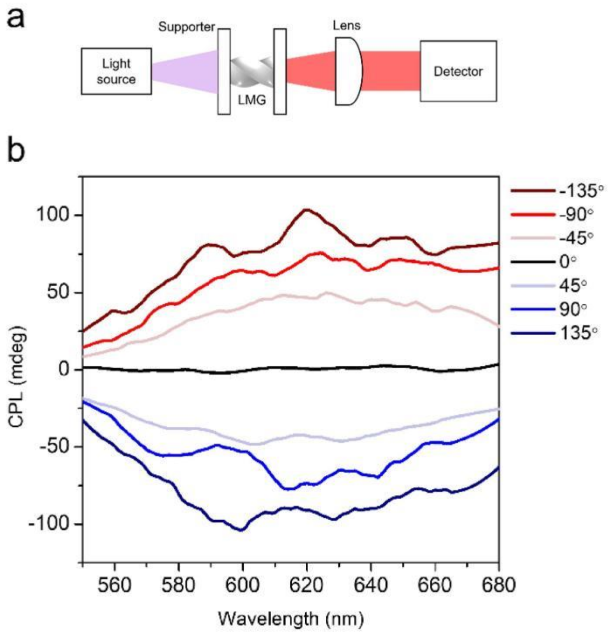

Figure R3–2. (a) Scheme of light path in the measurement of CPL and (b) CPL spectra of LMG depending on φ.

Comment 3: Does LMG present circular dichroism?

Our response: We thank the reviewer for raising this point. Yes, the twisted LMG exhibits circular dichroism (CD). While the luminophore itself is achiral, the twisted LMNP nanochain superstructures form a macroscopically chiral architecture that can generate chiroptical activity. Indeed, chiral superstructures assembled from magnetic nanomaterials under chiral magnetic fields have been reported to exhibit CD responses (*ACS Nano* **14**, 7152–7160 (2020); *Science* **380**, 1384–1390 (2023)).

To experimentally confirm CD in our system, we measured the CD spectra of LMGs at different

twisting angles φ. As shown in the new **Figure R3–3**, the CD response becomes increasingly pronounced as φ increases. This observation supports our interpretation that the measured CPL originates from the chiroptical response of the twisted LMG (i.e., CD–induced filtering of emitted light).

Importantly, we note that the measured CD sign is opposite to the measured CPL sign. This result is fully consistent with our interpretation that the observed CPL does not originate from intrinsic chiral emission of the luminophore, but instead arises from a CD–induced optical filtering mechanism. Specifically, when achiral photoluminescence propagates through the twisted LMG (acting as a chiral attenuator), the LCP and RCP components experience different attenuation due to the CD of the medium. Consequently, the transmitted/emerging emission becomes imbalanced between LCP and RCP, yielding an apparent CPL signal. Because CD measures the difference in extinction/absorbance between LCP and RCP, while CPL reflects the resulting difference in transmitted emission intensities after propagation, the CD and CPL can naturally exhibit opposite signs depending on the sign conventions of CD and CPL. Therefore, the observed opposite CD–CPL sign relation provides additional evidence supporting that the measured CPL is governed by the CD response of twisted LMGs.

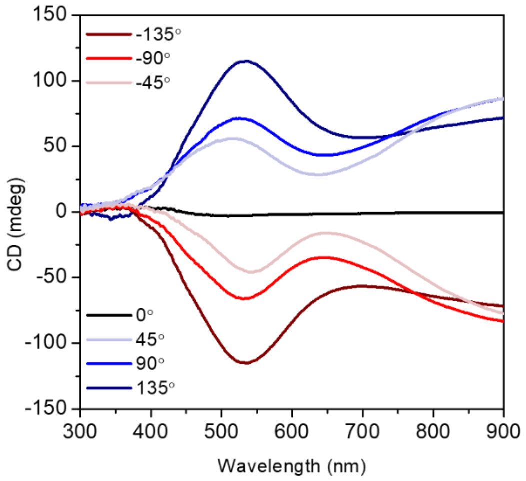

Figure R3–3. CD spectra of LMG under different φ.

Comment 4: The size of LMNP is about 300 nm. This is large enough to scatter lights in the LMG. How does it affect to CD and CPL in the LMG?

Our response: We sincerely thank the reviewer for this important and technically insightful comment. We agree that the LMNP size (~300 nm) places the system in the Mie scattering regime, and that scattering may affect the measured chiroptical responses of the twisted LMG. In a conventional Circular Dichroism (CD) spectrophotometer, the detector measures the intensity of light transmitted in the forward direction. The interaction between light and chiral

matter in CD setup is a balancing act between absorption and scattering (*Science* **365**, 1475–1478 (2019)). When dealing with nanomaterials or large aggregates, the CD is rarely just about electronic transitions.

A standard CD instrument measures the difference in the extinction of Left–Circularly Polarized (LCP) and Right–Circularly Polarized (RCP) light. Extinction (E) is physically defined as the sum of light lost due to absorption (A) and light lost due to scattering (S). Consequently, the measured CD signal (CD_{total}) is a combination of two distinct effects:

$$CD_{total} = (A_L - A_R) + (S_L - S_R)$$

The first term ($A_L - A_R$) represents the true Absorption CD, while the second term ($S_L - S_R$) is defined as Circular Differential Scattering (CDS) (*Proc. Natl. Acad. Sci. U.S.A.* **80**, 3568–3572 (1983)).

Nanoparticles and large aggregates are in the Mie scattering regime. In this regime, scattering becomes a dominant process because the particle size is comparable to the wavelength of the incident light. As the volume of the nanoparticle increases, its scattering cross–section grows significantly, often becoming much larger than its absorption cross–section (*Absorption and Scattering of Light by Small Particles* (Wiley–VCH, 2008)).

It is a common misconception that strong scattering automatically distorts CD. However, scattering only affects the CD signal if it is differential. If a large particle is achiral (e.g., a perfect gold sphere), it may scatter light intensely, but it will scatter LCP and RCP light with identical efficiency ($S_L = S_R$). In the subtraction process of the CD measurement, these equal scattering terms cancel out ($S_L - S_R = 0$). While this "non–differential" scattering can increase the noise in the data (due to fewer photons reaching the detector), it does not create a false CD peak (*Molecular Light Scattering and Optical Activity* (Cambridge University Press, 2004)).

For scattering to influence the CD spectrum, the structure must be both large and chiral. In chiral structures (such as nano–helices or twisted nanochains), the phase interference of light scattered from different parts of the structure depends on the handedness of the incident light. However, size and chirality are not the only requirements. The subunits must possess anisotropic polarizability (different response along different axes) or strong dipole–dipole coupling to distinguish between LCP and RCP light effectively (*J. Chem. Phys.* **76**, 3440–3446 (1982)).

When CDS is present, it introduces several characteristic artifacts into the CD spectrum that can lead to the misinterpretation of data (*ACS Photonics* **2**, 1602–1610 (2015)): (1) Non–Resonant Signals: CDS can generate "apparent CD" peaks in wavelength regions where the material does not actually absorb light (e.g., at wavelengths longer than the absorption edge). (2) Spectral Tailing and Broadening: Scattering signals are typically much broader than absorption bands and often manifest as long "tails" in the spectrum. (3) Sign Inversion and Peak

Shifts: Because CDS and Absorption CD are governed by different physical mechanisms, they can have opposite signs. Their superposition can shift the apparent peak position or even flip the sign of the measured CD, potentially leading to an incorrect assignment of the sample's handedness.

In our twisted LMG, scattering indeed exists because LMNP act as resonant/strongly scattering objects (**Figure R3–4a**). The extinction spectrum of LMG resembles that of LMNPs (**Figure R3–4b**).

To evaluate contribution of scattering–based chirality to the observed CPL experimentally, we measured circular differential scattering (CDS) by adapting the standard CD measurement geometry: instead of detecting extinction along the propagation direction, the detector was positioned orthogonally to the incident beam to selectively collect scattered light (**Figure R3–5a**). This approach allows us to isolate scattering dissymmetry (CDS) from extinction–based CD. The twisted LMG displays a measurable CDS spectrum; however, the CDS peak exhibits the opposite sign relative to the extinction CD (**Figure R3–5b**). This observation suggests that the scattering–induced chiral response would oppose, rather than reinforce, the helicity observed in CPL. Accordingly, CDS is unlikely to be the primary origin of the measured CPL, but rather a negative contribution to the measured CPL of the LMG. We therefore attribute the CPL mainly to the intrinsic chiroptical response reflected in the CD signal, while the influence of CDS on CPL is minor under our experimental conditions.

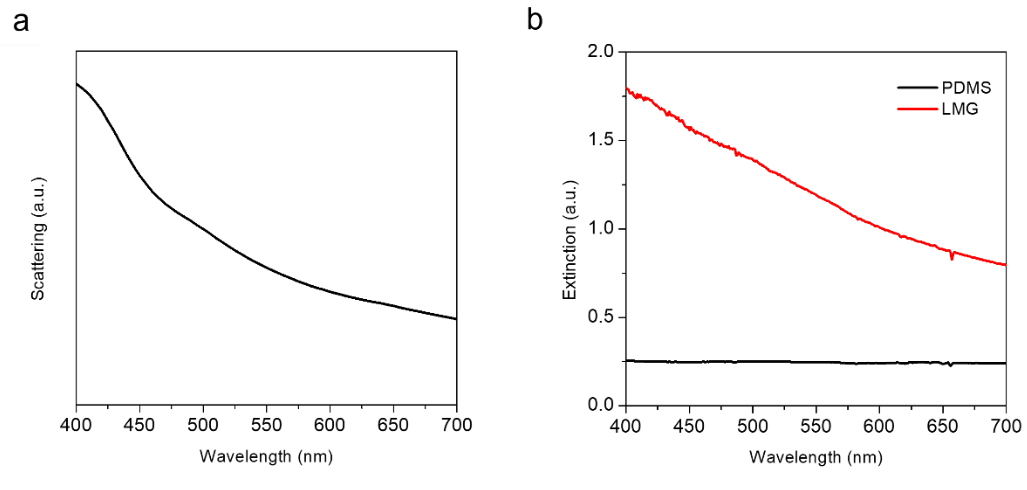

Figure R3–4. (a) Calculated scattering spectrum of twisted nanochain structures. (b) Measured extinction spectrum of PDMS and LMG.

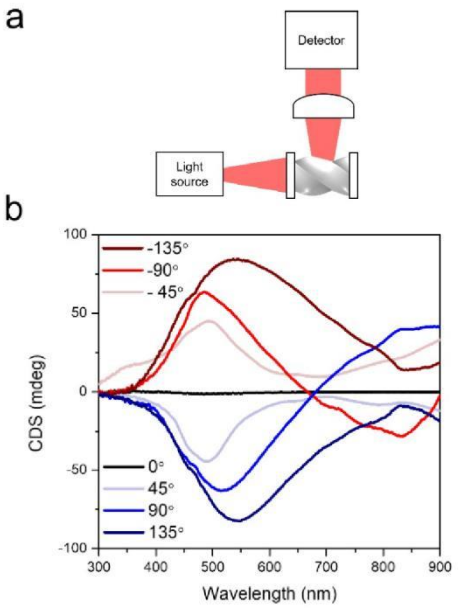

Figure R3–5. (a) Scheme of light path in the measurement of circular differential scattering (CDS) and (b) CDS spectrum of LMG depending on φ.

Comment 5: Under mechanical strains, PDMS experiences alignments of polymeric chains resulting in large birefringence. This can significantly affect to overall chiroptical responses. For instance, the Figure S11b shows stress–whitening of the samples, that indicate the optical responses already include birefringence from the polymeric chain alignments.

Our response: We thank the reviewer for raising this important point. We agree that PDMS can develop strain–induced chain alignment and birefringence under torsion, which in principle may introduce apparent chiroptical signals. We have therefore carefully evaluated whether the observed CPL originates from the chiral superstructures rather than from strain–induced PDMS birefringence.

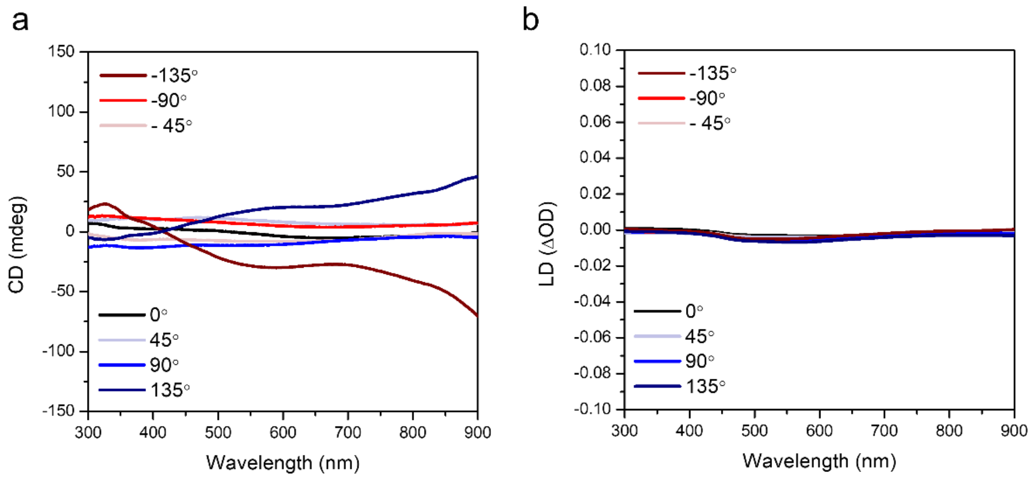

Figure R3–6. CD and LD spectra of bare PDMS depending on φ. (a) CD spectra and (b) LD spectra of bare PDMS without nanoparticles.

We have measured the CD and LD of bare PDMS without nanoparticles under twisting (**Figure R3–6**). As the reviewer pointed out, there was CD signal when it was twisted. But the CD intensity of bare PDMS was much weaker compared with that of LMG (**Figure R3–3**), and the twisting angle dependency was also not consistent. The LD was negligible in all twisting angle. Though bare PDMS shows CD under twisting, its weak intensity and lack of a clear angular dependence indicate that it cannot account for the dynamic CPL properties of LMG. We also provided the CPL spectra of LMG without magnetic assembly (Supplementary Fig.24), which does not show any CPL peaks. These data demonstrate that the twisting–induced CPL does not originate from the deformation–induced polymeric matrix birefringence, but arises from the assembled superstructures in LMG.

We would also like to politely point out that, while in general PDMS can experience polymer chain alignment under mechanical strains, the white appearance of the sample surface in Figure S11b (modified as Figure S15b in the revised supporting information) is most probably an optical artifact caused by light reflection at the camera's viewpoint, rather than the stress–induced polymeric chain alignment. This is supported by two observations: first, the shadow sample region (near the upper specimen) does not show surface whiteness, and second, the specimen surface whiteness does not change with increasing strain.

Comment 6: Twisting can induce the breakage of the nanoparticle chain alignments beside the helical alignments. The degree of breakage might become greater in greater degree of twist. This may destroy helical shaped alignments? Are there any experimental data?

Our response: We sincerely appreciate the reviewer's thoughtful question regarding the possibility of nanoparticle chain breakage during the twisting process.

In our system, twisting primarily induces an angular displacement around the z–axis, rather than significant deformation along the x– or y–dimensions where the LMNP nanochains are oriented (**Figure R3–7**). Because the nanochains lie within the x–y plane, and because the projected area of the x–y plane remains nearly constant before and after twisting, the mechanical strain applied to the nanochains is predominantly rotational rather than tensile or compressive. Consequently, the probability of chain breakage is substantially lower than what would be expected if the twisting imposed significant elongation or compression within the plane. In addition, the reversible and repeatable CPL modulation supports that the nanochain structures can be maintained after mechanical deformation.

To provide experimental evidence, we have conducted confocal microscope observation before and after twisting (**Figure R3–8**). Even the repeated twisting cycles, the assembled nanochain structures maintained their linear structures without outstanding breakage.

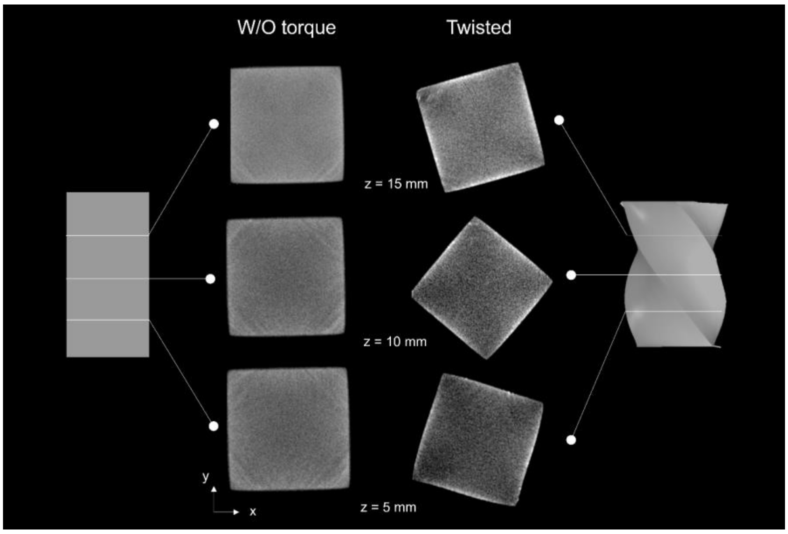

Figure R3–7. CT cross section (x–y plane) images of LMG with different z–positions. The z–position of each CT cross section images is marked at the corresponding LMG scheme.

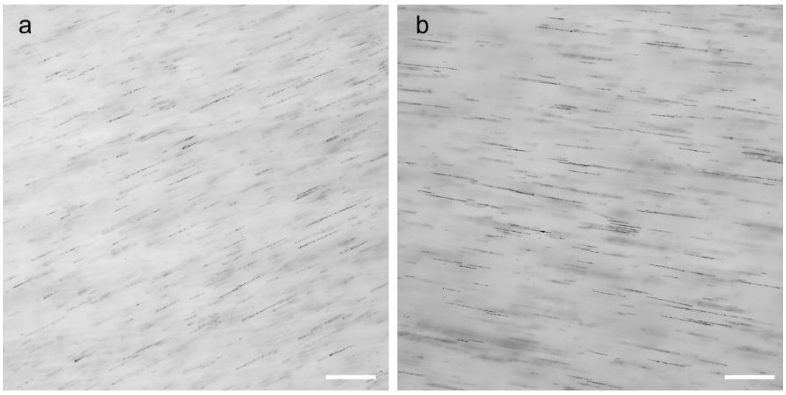

Figure R3–8. Confocal microscope images of assembled nanochain structures (a) before and (b) after tens of twisting cycles. Scale bars are 50 μm.

Our revision: To answer the reviewer's important comment, we have provided confocal microscope images of the assembled nanochain structures before and after twisting (Figure R3–8) in the revised supplementary information as Supplementary Fig. 14 and added corresponding description "Even after repeated twisting, the structures maintained their linear configurations without breakage (Supplementary Fig. 14)." on Page 9, line 3–5, in the revised manuscript.

For Reviewer #1:

Comment: The manuscript has been significantly improved after the authors carefully addressed all the reviewers' question and concerns. Thus, i recommend the acceptance of this work to be published in Nature Communications.

Our response: We sincerely thank the reviewer for the positive evaluation and for recommending the acceptance of our work. We are grateful for the reviewer's insightful comments, which have helped us significantly improve the quality and clarity of the manuscript.

For Reviewer #2:

Comment: I thank the authors for considering my comments and providing the revised manuscript. All my concerns have been addressed properly and the revised manuscript is sutiable for publication.

Our response: We sincerely appreciate the reviewer's constructive comments throughout the revision process, which have greatly improved the quality of our manuscript.

For Reviewer #3:

General comment: The authors have satisfactorily addressed all of the reviewers' comments. I appreciate the additional experiments and the thorough explanations provided in the Response letter. As a suggestion, if feasible, the authors may consider including some of the key points from the discussion made on Reviewer 3, Comments 2~5 (Figures R3-2 to R3-6), at least in the Supporting Information. This would provide readers with a more comprehensive understanding of the CD and scattering effects in the mechanically strained systems and would be valuable to researchers in the related field.

Our response: We would like to thank the reviewer for the insightful suggestion. We agree that including the detailed discussions on CD and scattering effects will be highly beneficial for readers and researchers in the field.

Our revision: Following the reviewer's helpful suggestion, we have made the following revisions in both the revised manuscript and the revised supplementary information:

Figures R3-2, R3-3, R3-4, R3-5 and R3-6 have been added in the revised supplementary information as Supplementary Fig.24, 25, 34, 35 and 36, incorporating with the key discussions to provide more comprehensive understanding of the chiroptical properties of our system. Corresponding description "Other factors such as illumination direction and matrix effect showed similar results (Supplementary Figs.24 and 25)." has been added and highlighted in the revised manuscript on page 7, line 14-15; "Supplementary Fig.34-36" has been added and highlighted in the revised manuscript on page 8, line 14.
